# Performance difference of graph-based and alignment-based hybrid error correction methods for error-prone long reads

**DOI:** 10.1186/s13059-019-1885-y

**Published:** 2020-01-17

**Authors:** Anqi Wang, Kin Fai Au

**Affiliations:** 1 Department of Biomedical Informatics, The Ohio State University, Columbus, OH 43210 USA; 2 Department of Internal Medicine, University of Iowa, Iowa City, IA 52242 USA; 3 Department of Biostatistics, University of Iowa, Iowa City, IA 52242 USA

## Abstract

The error-prone third-generation sequencing (TGS) long reads can be corrected by the high-quality second-generation sequencing (SGS) short reads, which is referred to as hybrid error correction. We here investigate the influences of the principal algorithmic factors of two major types of hybrid error correction methods by mathematical modeling and analysis on both simulated and real data. Our study reveals the distribution of accuracy gain with respect to the original long read error rate. We also demonstrate that the original error rate of 19% is the limit for perfect correction, beyond which long reads are too error-prone to be corrected by these methods.

## Background

Third-generation sequencing (TGS) technologies [1], including Pacific Biosciences (PacBio) and Oxford Nanopore Technologies (ONT), have been demonstrated useful in many biomedical research since the unprecedented read lengths (average for PacBio and ONT can be over 10 kb and 20 kb, and maximum over 60 kb and 800 kb) are very informative for addressing complex problems, such as genome assembly and haplotyping [1–10]. However, the high error rates of TGS data (average 10–15% for the raw data) [11–14] reduce the mappability and the resolution of downstream analysis. To address this limitation, the high-quality short reads have been used to correct the long reads, which is termed as hybrid error correction. The existing hybrid error correction methods can be classified into two categories: alignment-based method [15–21] and de Bruijn graph (DBG)-based method (referred as “graph-based method”) [22–26]. Regardless of the lower algorithmic complexity by the graph-based method than the alignment-based one [27] and the difference of software implementations, several principal factors have significant effects on the error correction performance for both methods: long read error rate, short read error rate, short read coverage, alignment criterion, and solid *k*-mer size. Although previous studies examined some of these factors separately in the corresponding software development [28–30], here we establish mathematical frameworks to perform a comprehensive investigation of all these factors in hybrid error correction. Through studying their influences on short read alignment rate and solid *k*-mer detection in DBG, we finally interrogate how these factors determinate the accuracy gain in hybrid error correction. This research does not only study the algorithmic frameworks of two major hybrid error correction methods, more importantly it also offers an informative guidance for method selection, parameter design, and future method development for long read error correction.

## Results and discussion

Overall, we first evaluate the accuracy gains by the alignment-based and graph-based methods at each error rate level by mathematical modeling, following by validating the model fitness with simulated and real data. With these data and results, we study the influences of key algorithmic factors under different data scenarios, and compare two methods.

**Fig. 1:**
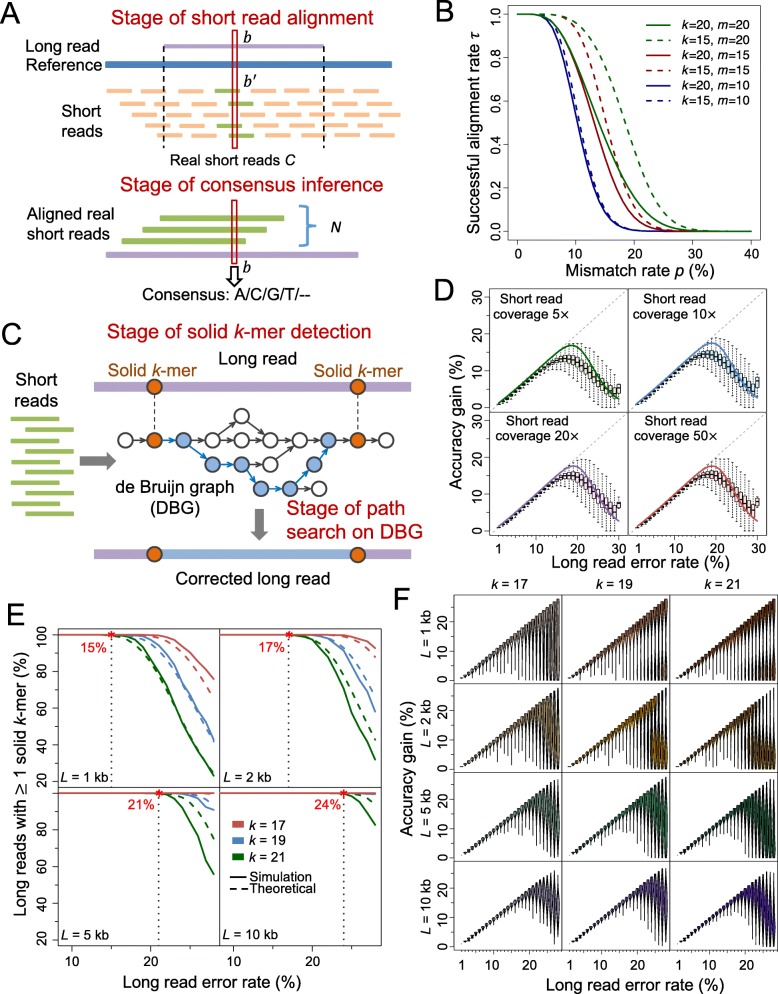
Illustration of alignment-based and graph-based method; results for model fitness and accuracy gain on simulated data. **a** Schematic of alignment-based method. *b* is a certain base on the long read, and *b*^′^ is the corresponding base on the reference sequence. The *C* real short reads are aligned to the long read (with *N* of them being successfully aligned), and then the consensus is inferred at each base. **b** Relationship of the successful alignment probability for short reads *τ* with the mismatch rate *p*, lower threshold on perfect match *k*-mer size *k* and the upper threshold of mismatches *m*. In spite of the changes of *k* or/and *m*, *τ* is near to one when *p* < 5%, and is near to zero when *p* > 30%. This indicates that mismatch rate is the most dominant factor on *τ*. As *m* increases from 10 to 20, the curves move upper (from blue to red and green), implying that *τ* increases with *m*. Moreover, the divergence between the dashed and solid blue, red, and green lines also shows an increasing tendency, which means the effect of *k* on *τ* also increases with *m*. **c** Schematic of graph-based error correction method. DBG is built based on short reads. Solid *k*-mers are detected on the long reads. The fragment between two adjacent solid *k*-mers is then aligned with the correlated path on the DBG. The path is used to correct the fragment when certain criteria are satisfied. **d** Accuracy gain at each error rate for simulated long reads corrected by alignment-based method. The boxplots represent the accuracy gain distribution for long reads. The solid lines represent the theoretical values. The dashed gray lines (diagonal lines) correspond to perfect correction. **e** Proportion of simulated long reads with solid *k*-mer detected at each error rate level. The solid lines represent the theoretical values. The dashed lines represent the results on simulated long reads. **f** Accuracy gain at each error rate for simulated long reads corrected by graph-based method. *L*: long read length; *k*: size of perfectly matched seed or solid *k*-mer

Two major stages of the alignment-based method determine the accuracy gain: short read alignment and consensus inference (Fig. 1a). Denote *C* as the number of short reads generated at a certain base in sequencing process, which is referred as the real short reads. At the first stage, the *C* real short reads are aligned to the long reads. Let *N* be the number of successfully aligned real short reads. Next, per the base of interest, the consensus of the aligned real short reads is generated as the corrected base. We define accuracy gain as *γ* − (1 − *EA*), where *γ* is the original long read error rate and *EA* is the expected accuracy after error correction:

$$ EA=\sum \limits_{n=0}^C\Pr \left(N=n\right)g\left(n,\beta \right). $$

Pr(*N* = *n*) represents the probability that *n* real short read can be successfully aligned, corresponding to the stage of short read alignment, and *g*(*n*, *β*) is the probability that the consensus equals to the true base, corresponding to the stage of consensus inference. *β* is the short read error rate. At first we calculate Pr(*N* = *n*) via obtaining the probability of successfully aligning a single short read to long read, which highly depends on the tolerance of mismatches and the length of perfectly matched seed required by an aligner. For two sequences *X* and *Y* with equal length *l*, denote *M* as the number of mismatched bases, and *K* as the length of the largest perfectly matched seed. Let *k* be a lower threshold of *K*, and *m* be an upper threshold of *M* and thus the couple of conditions *K* ≥ *k* and *M* ≤ *m* sets up a criterion of alignment. The following theorem measures the probability *τ* that a single short read can be successfully aligned under the criterion.

**Theorem 1.** *Let X and Y be two sequences with equal length l. Denote X*_*i*_ *and Y*_*i*_ (1 ≤ *i* ≤ *l*) *as the i*^*th*^ *bases of X and Y, respectively. Suppose all the events* {*X*_*i*_ = *Y*_*i*_} *are independent, and all the bases have a common mismatch rate p. Let τ*(*k*, *m*, *p*, *l*) ≜ Pr(*K* ≥ *k*, *M* ≤ *m*)*,* 0 ≤ *m* ≤ *l, where τ is namely the probability that a short read can be successfully aligned to a target place on the long read by an aligner requiring a perfectly matched seed not shorter than k and the number of mismatched bases not more than m. We have:*

$$ \tau \left(k,m,p,l\right)=\sum \limits_{n=0}^m\left[\sum \limits_{t=1}^{Q(n)}{\left(-1\right)}^{t-1}\left(\begin{array}{c}n+1\\ {}t\end{array}\right)\left(\begin{array}{c}l- kt\\ {}n\end{array}\right)\right]{p}^n{\left(1-p\right)}^{l-n}, $$

*where Q*(*n*) = max {*s*| *l* − *ks* ≥ *n*} ⋀ (*n* + 1)*. τ increases with m and l, and decreases with k and p****.***

The proof is provided in Additional file 1: Note 1. Based on *τ*, we are able to calculate the alignment rate of *N* short reads Pr(*N* = *n*). Given a set of errors in a long read, alignments of short reads are not completely independent, so we consider short reads in several batches (Additional file 1: Note 2, Figure S1). The mismatch rate *p* can roughly be estimated by *β* + *γ* (Additional file 1: Note 3). The analytical results indicate that the mismatch rate (i.e., approximately the long read error rate, because *β* ≪ *γ*), is the most dominant factor on *τ*; as *m* increases, both *τ* and the effect of *k* on *τ* increase (Fig. 1b, Additional file 1: Note 4). The accuracy of consensus inference *g*(*n*, *β*) can be deducted based on binomial distribution (Methods, Additional file 1: Note 5). The theoretical calculation shows that shallow aligned short read coverage is enough to generate high-accuracy consensus (e.g., only 9× aligned short reads can achieve consensus with accuracy >99.99%), so short read alignment is the dominant stage that impacts accuracy gain (Additional file 1: Figure S2).

Two stages in the graph-based method, including detection of solid *k*-mer and path search in DBG, influence the accuracy gain (Fig. 1c). At the first stage, all *k*-mers on the long read are scanned to find the “solid *k*-mers” that exist in the DBG generated by short reads. At the second stage, all paths that link two adjacent solid *k*-mers or link a solid *k*-mer with the end of long read on the DBG are searched to find the optimal one to correct the long read. Let φ(*k*, *γ*, *L*) be the probability that the long read (with length *L*) contains at least one solid *k*-mer. According to Theorem 1, we have:

$$ \varphi \left(k,\gamma, L\right)=\tau \left(k,L-k,\gamma, L\right)=\sum \limits_{n=0}^{L-k}\left[\sum \limits_{t=1}^{Q(n)}{\left(-1\right)}^{t-1}\left(\begin{array}{c}n+1\\ {}t\end{array}\right)\left(\begin{array}{c}L- kt\\ {}n\end{array}\right)\right]{\gamma}^n{\left(1-\gamma \right)}^{L-n} $$

(see Methods, Additional file 1: Note 6, Figure S3 for details). To investigate the second stage, we examine the distance between adjacent solid regions, since it represents the overall difficulty of path search in DBG. We model the solid region distance by a truncated geometric distribution compounded with a geometric distribution, and its expectation increases with *k*-mer size *k* and long read error rate *γ* (see Methods for details).

Next, we examine the model fitness and accuracy gains of both methods on simulated data. The long reads and short reads are simulated from the *E. coli* reference genome (strain K-12 MG1655) (Additional file 1: Note 7) [31, 32]. The alignment-based software proovread [19] is applied to correct the long reads (Additional file 1: Note 8, Figure S4). The tendencies of the theoretical accuracy gains fit the actual accuracy gains on the simulated data under different short read coverages (Fig. 1d). When *γ* ≤ 15%, even if very shallow short read coverage is used (5×), the accuracy gain increases along the diagonal line, which implies nearly perfect correction. When *γ* ≥ 18%, the accuracy gain decreases and the corresponding variance increases, and thus very few reads can be perfectly corrected. These results show the upper limit of long read error rate that the alignment-based method can perfectly solve, and the similar results are demonstrated in the graph-based method (as shown below). Moreover, both theoretical calculation and simulated data reveal that the accuracy gain can rarely exceed 20%, although there is slight increment (e.g., <2% and <1%) with respect to short read coverage (e.g., from 5× to 10× and from 20× to 50×, respectively, Fig. 1d). Therefore, the hybrid error correction benefit marginally from increase of short read coverage, especially when it is greater than 10×.

To evaluate the model of graph-based method, we apply LoRDEC (version 0.5.3) [23] to correct the simulated long reads (Additional file 1: Note 9). The short read coverage is 10× in this evaluation. The overall tendencies of the theoretical solid *k*-mer detection rate φ with respect to the length of long read *L* and the required *k*-mer size *k* align well with the values generated from the simulated data (Fig. 1e), though φ is slightly higher when *L* is over 2 kb. Overall, the solid *k*-mer detection rate is close to 1 when long read error rate *γ* is below certain threshold (such as 15% for *k* = 21 and *L* = 1 kb), and it decreases dramatically as *γ* increases beyond the threshold. This threshold increase with *L* (e.g., from 15% to 24% for 1 to 10 kb given *k* = 21) (Fig. 1e). In addition, the increase of *k*-mer size has an overall negative effect on solid *k*-mer detection, which is more remarkable when long reads are shorter (Fig. 1e). Of note, high long read error rate results in high probability that no solid *k*-mer can be detected so that the long read cannot be corrected. Following solid *k*-mer detection, we investigate the distances between adjacent solid regions: for all *k*-mer sizes in the test, the theoretical distances are consistent with the actual values obtained in the simulated data at different levels of long read error rates (Additional file 1: Figure S5). Given a *k*-mer size, both the mean and variance of the distances increase remarkably when long read error rate is ≥18% while it rarely exceeds 500 bp otherwise (Additional file 1: Figure S5). In addition, the increase of *k* also leads to a substantial increment on the distance.

In term of accuracy gain, the simulated data show that long reads can be almost perfectly corrected by the graph-based method when the long read error rate *γ* ≤ 19%, and the accuracy gain decreases and the corresponding variance increases when *γ* > 19%. The corresponding change point of *γ* in the alignment-based method is ~ 15%. However, instead of a single peak of accuracy gain with respect to *γ*, there is a bimodal pattern with *γ* > 19% in some scenarios of the graph-based method (e.g., *k* ≥ 19 and *L* ≤ 2 kb): some long reads can be corrected almost perfectly while some others have zero or very low accuracy gain (Fig. 1f). The latter subset of long reads may likely contain no or only one solid *k*-mer, so no or very difficult correction is performed. When the length of long read *L* increases to ≥5 kb, the distribution of accuracy gain shrinks at every error rate level and the bimodal pattern fades. Because longer read length improves the probability of solid *k*-mer detection (see the abovementioned results and Fig. 1e), a larger proportion of long reads can be corrected even though not perfectly.

**Fig. 2:**
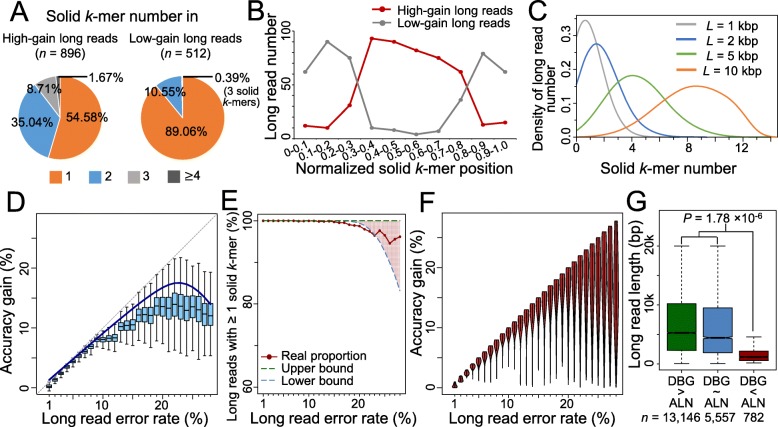
Explanation of bimodal accuracy gain for graph-based method; model fitness and accuracy gain on real dataset. **a** Proportion of long reads with different solid *k*-mer number. Without loss of generosity, the simulated long reads with length of 1 kb and error rate of 25% are taken as example. A long read is labeled as “high-gain long read” of the accuracy gain is larger than 12.5% (half of the value of error rate), and “low-gain long read” otherwise. **b** Distribution of the single solid *k*-mer locations on the high-gain and low-gain long reads. Only the long reads with one solid *k*-mer are considered. **c** Distribution of solid *k*-mer number on the long reads with different lengths. **d** Accuracy gain distribution at each error rate level for alignment-based method. **e** Proportion of long reads with solid *k*-mer detected. Due to the mixture of different long read lengths, an upper boundary and lower boundary is provided. **f** Accuracy gain distribution at each error rate level for graph-based method. **g** Length distribution of long reads on which graph-based method (labeled as DBG) has better, equal, or worse performance than the alignment-based method (labeled as ALN). The *p* value is calculated by Wilcoxon rank sum test

The bimodal pattern of accuracy gain is further investigated through a concrete scenario, in which *k* = 19, *L* = 1 kb, *γ* = 25%. The corrected reads are classified into two groups: “high-gain long reads” with accuracy gain >12.5%, and “low-gain long reads” otherwise. Much higher fraction of the low-gain long reads contains only one solid *19*-mer than the high-gain long reads (89.04% vs. 54.58%, Fig. 2a), and overall, the former contain more solid *19*-mers than the latter. Moreover, for long reads with single *19*-mer, the locations of the *19*-mers are different for two classes of long reads: at the middle of high-gain long reads, while near either end of low-gain long reads (Fig. 2b). When the solid *k*-mer occurs near an end of the long read, one fragment is particularly long so that the correction by path search in DBG becomes more difficult, resulting in lower accuracy gain. In the case that no solid *19*-mer is detected, long reads are uncorrected and contribute to the modal with low accuracy again as well. As the read length increases, more reads contain multiple solid *19*-mer (Fig. 2c) and the effect of fragments at the ends becomes marginal so that the bimodal pattern disappears.

We further study the accuracy gains on a real PacBio dataset [23] corrected by proovread and LoRDEC, respectively (Additional file 1: Note 8–10, Figure S6, Figure S7). Short reads are randomly sampled with coverage 10×. The overall tendency of the real accuracy gain by proovread is in accordance with the theoretical calculation of the alignment-based method, though there is slight overestimation by the latter (Fig. 2d). On the real data, long reads can rarely obtain accuracy gain >20% (Fig. 2d). However, when the long read error rate increases from 25 to 30%, the accuracy gain maintains at a range of 10–15% rather than showing a sharp decrease as the theoretical modeling. When evaluating the accuracy gain by LoRDEC on the real data, it should be noticed that the real data contains long reads with different lengths, in contrast to the fixed read length in the abovementioned mathematical model of the graph-based method. Despite this difference, the proportion of the real long reads with solid *k*-mer detected is within the theoretical range (Fig. 2e), and the pattern of accuracy gain is very similar with the simulated results (Fig. 2f and Fig. 1f): most long reads achieve nearly perfect correction when the error rate is <20%, and the variance becomes larger for higher error rates.

Furthermore, two methods are compared based on the real dataset. The difference of accuracy gains between two methods becomes remarkable when the long read error rate >15%. Among 19,485 long reads with original error rates >15%, LoRDEC outperforms proovread on 13,146 (67.47%) reads, i.e., the difference of accuracy gains is >2% (boxplots in Fig. 2d vs. violin plots in Fig. 2f). Two methods show similar accuracy gains in 5,557 (28.52%) long reads, i.e., the difference of accuracy gains is ≤2%. proovread performs better for the remaining 782 (4.01%) reads. The third group of long reads is significantly shorter than the other two groups (*p* value of Wilcoxon rank sum test 1.78 × 10^−6^, Fig. 2g). It is consistent with the abovementioned inference: for the graph-based method, shorter reads are more likely to contain few or no solid *k*-mers, and the location of the solid *k*-mer highly affects the correction (Fig. 2a–c).

In summary, the theoretical calculation by mathematical frameworks together with both analyses of simulated and real data shows how key algorithmic factors and data parameters affect the accuracy gains by two main types of hybrid error correction algorithms. When the original long read error rate is below certain thresholds (e.g., 15%), both methods can correct most errors. For highly error-prone long reads (especially *γ* ≥ 20%), the graph-based method can obtain generally higher accuracy gain, while the variance is also larger. Among such highly error-prone long reads, the alignment-based method tends to have more advantage in correcting relatively shorter ones (e.g., median length 1,195 bp in our test, Fig. 2g). Although it is not possible to analyze all published software, the results generated by proovread and LoRDEC are representative for the alignment-based and graph-based methods, respectively, as shown by our previous benchmark work on 10 error correction software [27]. Of note, sequencing errors along real long reads may not be independent, or short read coverage may not be evenly distributed (e.g., transcriptome data), so specific adjustment is necessary in the analysis of real data (see Additional file 1: Note 10–11 for details). As both PacBio and ONT improve the technologies, the error rates of most raw data become <20%. At this range, our results fit the real data very well and thus will be beneficial for the analyses of the real data and provide a guidance for method selection, parameter design (Additional file 1: Note 12–13, Figure S8) and future method development. In addition, for modeling the alignment-based method, the mathematical theorem is established to measure the probability of short read alignment, which also lays the groundwork of development and analyses of the other alignment-based algorithms.

## Methods

### Model for consensus inference in an alignment-based method

The model for short read alignment, which is the first stage in alignment-base method, has been shown above with Theorem 1. Next, at consensus inference stage, the base with ≥50% frequency is taken as consensus. Thus, the main factors that influence consensus accuracy are short read error rate and the number of aligned short reads.

Let *a* be the real base on a certain site of a long read. Denote $\mathcal{V}=\left\{{V}_1,{V}_2,\cdots, {V}_N\right\}$ as the corresponding bases on the *N* aligned short reads. Thus, Pr(*V*_*i*_ = *a*) = 1 − *β*, where *β* is the short read error rate. Let $F\left(\mathcal{V}\right)$ be the consensus function:

$$ F\left(\mathcal{V}\right)=\underset{s\in \left\{A,C,G,T,-\right\}}{\mathrm{argmax}}{\sum}_{i=1}^NI\left({V}_i=s\right). $$

*I*(∙) is the indicator function. Considering the half-vote criterion, we have

$$ \Pr \left(F\left(\mathcal{V}\right)=a\right)\ge \Pr \left({\sum}_{i=1}^NI\left({V}_i=a\right)\ge \left\lceil \frac{N}{2}\right\rceil \right)\triangleq g\left(N,\beta \right). $$

*g*(*N*, *β*) is the accuracy of consensus inference and is defined as:

$$ g\left(N,\beta \right)=\Pr \left({W}_{N,1-\beta }>\frac{N-1}{2}\right),N\  is\  odd. $$

$$ g\left(N,\beta \right)=\Pr \left({W}_{N,1-\beta }>\frac{N}{2}\right)+\frac{1}{2}\Pr \left({W}_{N,1-\beta }=\frac{N}{2}\right),N\  is\ even. $$

*W*_*N*, *β*_ follows the binomial distribution Binom(*N*, 1 − *β*). It can be proved that *g*(*N*, *β*) increases with *N* and decreases with *β* (See the two lemmas and detailed results in Additional file 1: Note 5).

### Model for solid *k*-mer detection in graph-based method

The solid *k*-mer detection requires that (1) the long read contains continuous *k* error-free bases; (2) the *k*-mer is also present in the DBG. Because of the high accuracy of short reads, the condition (2) is very likely guaranteed even with shallow short read coverage (Additional file 1: Note 6). Below we calculate the probability of (1). Suppose all bases on the long read are independent with a common error rate *γ*. Denote the probability that the long read contains at least one correct *k*-mer as φ(*k*, *γ*, *L*) ≜ Pr(*K* ≥ *k*). According to Theorem 1,

$$ \varphi \left(k,\gamma, L\right)=\tau \left(k,L-k,\gamma, L\right)={\sum}_{n=0}^{L-k}\left[{\sum}_{t=1}^{Q(n)}{\left(-1\right)}^{t-1}\left(\begin{array}{c}n+1\\ {}t\end{array}\right)\left(\begin{array}{c}L- kt\\ {}n\end{array}\right)\right]{\left(1-\gamma \right)}^{L-n}. $$

φ(*k*, *γ*, *L*) decreases with *k* and *γ*, and increases with *L*. In contrast to the application of Theorem 1 with fixed read length of short reads *l* in alignment-based methods, the application of Theorem 1 in a graph-based method uses the length of long reads *L*, which is variable and substantially larger.

### Model for solid region distance in a graph-based method

Denote *S* as the distance between adjacent solid regions, and *T* as the length of the maximal correct segment which is smaller than *k*. It has a probability function

$$ \Pr \left(T=t\right)=\frac{{\left(1-\gamma \right)}^t\gamma }{1-\alpha }, $$

where

$$ \alpha ={\sum}_{t=k}^{\infty }{\left(1-\gamma \right)}^t\gamma . $$

*α* is the probability that at least *k* continuous bases on the long read are correct. Suppose {*T*_*i*_; *i* ≥ 1} are independent observations of *T*, then we have

$$ S={\sum}_{i=1}^N{T}_i+N-1. $$

where *N* is the number of maximal correct segments between the solid regions and it follows a geometric distribution,

Pr(*N* = *n*) = (1 − *α*)^*n*^*α*, *n* ≥ 0.

The expectation of *S* is

$$ ES=E\left(E\left(S|N\right)\right)=E\left(N\left( ET+1\right)\right)-1=\left( ET+1\right) EN-1. $$

The expectation of solid region distance increases with *k* and *γ*.

### Real data, data simulation, data processing, and software usage

The simulated long reads and short reads are generated by SimLoRD [31] and ART [32], respectively (see Additional file 1: Note 7 for details). The typical alignment-based and graph-based software, proovread [19] and LoRDEC [23], are used to correct the long reads (Additional file 1: Note 8–9). The details for processing real data can be found in Additional file 1: Note 10.

## Supplementary information

**Additional file 1: Note 1.** Proof of Theorem 1. **Note 2.** Dependence of short read alignment. **Note 3.** Estimation of mismatch rate *p*. **Note 4.** Relationship between *τ* and parameters *p*, *k*, *m*. **Note 5.** Monotonicity of consensus inference accuracy. **Note 6.** Probability that a *k*-mer is present in DBG. **Note 7.** Generation of simulated data **Note 8.** Application of proovread. **Note 9.** Application of LoRDEC. **Note 10.** Processing of real data. **Note 11.** Model application to transcriptome sequencing data. **Note 12.** Suggestion on method selection. **Note 13.** Parameter design for organisms with different genome complexity. **Figure S1.** Independence of short read alignment and the related model. **Figure S2.** Relationship between consensus accuracy and short read coverage. **Figure S3.** Relationship between short read coverage and the probability that a solid *k*-mer is presend in the DBG created by short reads. **Figure S4.** Selection of mismatch threshold *m* for the model of alignment-based method. **Figure S5.** Relationship between adjacent solid region distance and long read error rate. **Figure S6.** Relationship between long read error rate and theoretical rate of the fitted geometric distribution. **Figure S7.** Relationship between long read error rate and proportion on long read with short reads being aligned. **Figure S8.** *k*-mer specificity of the genomes from different organisms.

**Additional file 2.** Review history.

## Data Availability

The Illumina and PacBio sequencing data of *E. coli* are downloaded from Sequence Read Archive: ERR022075, and PacificBiosciences/DevNet (https://github.com/PacificBiosciences/DevNet/wiki/E.-coli-Bacterial-Assembly) [23]. The simulated data are uploaded to NCBI under the project PRJNA574878 [33].
