# Supplementary material for: Performance difference of graph-based and alignment-based hybrid error correction methods for error-prone long reads
Source: Genome Biol. 2020 Jan 17;21:14. doi: 10.1186/s13059-019-1885-y (PMC6966875; doi:10.1186/s13059-019-1885-y)
Supplement: Supplementary file 1 — Additional file 1: Note 1. Proof of Theorem 1. Note 2. Dependence of short read alignment. Note 3. Estimation of mismatch rate p. Note 4. Relationship between τ and parameters p, k, m. Note 5. Monotonicity of consensus inference accuracy. Note 6. Probability that a k-mer is present in DBG. Note 7. Generation of simulated data Note 8. Application of proovread. Note 9. Application of LoRDEC. Note 10. Processing of real data. Note 11. Model application to transcriptome sequencing data. Note 12. Suggestion on method selection. Note 13. Parameter design for organisms with different genome complexity. Figure S1. Independence of short read alignment and the related model. Figure S2. Relationship between consensus accuracy and short read coverage. Figure S3. Relationship between short read coverage and the probability that a solid k-mer is presend in the DBG created by short reads. Figure S4. Selection of mismatch threshold m for the model of alignment-based method. Figure S5. Relationship between adjacent solid region distance and long read error rate. Figure S6. Relationship between long read error rate and theoretical rate of the fitted geometric distribution. Figure S7. Relationship between long read error rate and proportion on long read with short reads being aligned. Figure S8. k-mer specificity of the genomes from different organisms. [file 13059_2019_1885_MOESM1_ESM.pdf]

Additional Notes

1 Proof of Theorem 1

Proof. According to the total probability formula:

$$\Pr(K \geq k, M \leq m) = \sum_{n=0}^{m} \Pr(K \geq k | M = n) \Pr(M = n) \tag{1}$$

The assumptions imply that M follows binomial distribution $\text{Binom}(l, p)$, so that

$$\Pr(M = n) = \binom{l}{n} p^n (1-p)^{l-n} \tag{2}$$

When $n (\geq 1)$ mismatches occur between X and Y, we denote the indices of the mismatched base-pairs as $\Lambda_1, \cdots, \Lambda_n (\Lambda_1 < \cdots < \Lambda_n)$, which means $X_{\Lambda_1} \neq Y_{\Lambda_1}, \cdots, X_{\Lambda_n} \neq Y_{\Lambda_n}$. The n mismatched base-pairs divide X and Y into $n+1$ parts, and we denote the length of each perfectly matched subsequence as

$$Z_1 = \Lambda_1 - 1, Z_2 = \Lambda_2 - \Lambda_1 - 1, \cdots, Z_n = \Lambda_n - \Lambda_{n-1} - 1, Z_{n+1} = l - \Lambda_n.$$

Then we have

$$\Pr(K \geq k | M = n) = \Pr\left(\bigcup_{j=1}^{n+1} \{Z_j \geq k\}\right) = \frac{\#\left(\bigcup_{j=1}^{n+1} \{Z_j \geq k\}\right)}{\binom{l}{n}} \tag{3}$$

According to the inclusion exclusion principal,

$$\#\left(\bigcup_{j=1}^{n+1} \{Z_j \geq k\}\right) = \sum_{s=1}^{n+1} (-1)^{s-1} \sum_{i_1 < \cdots < i_s} \#\left(\bigcap_{t=1}^{s} \{Z_{i_t} \geq k\}\right).$$

Given $i_1, i_2, ..., i_s$, we define two $(n+1)$-dimensional sets $\mathcal{Z}_{i_1, i_2, ..., i_s}$ and $\mathcal{W}_{i_1, i_2, ..., i_s}$,

$$\mathcal{Z}_{i_1, i_2, ..., i_s} = \{(z_1, z_2, ..., z_n, z_{n+1}) | z_j \in \mathbb{Z}, z_j \geq 0, \sum_{j=1}^{n+1} z_j = l - n, z_{i_1} \geq k, ..., z_{i_s} \geq k\},$$

$$\mathcal{W}_{i_1, i_2, ..., i_s} = \{(w_1, w_2, ..., w_n, w_{n+1}) | w_j \in \mathbb{Z}, w_j \geq 0, \sum_{j=1}^{n+1} w_j = l - n - ks\}.$$

It can be seen that

$$\#\left(\bigcap_{t=1}^{s}\{Z_{i_t}\geq k\}\right)=\#(\mathcal{Z}_{i_1,i_2,\dots,i_s}).$$

Next, we define a mapping $f_{i_1,i_2,\dots,i_s}$ as

$$f_{i_1,i_2,\dots,i_s}:\mathcal{Z}_{i_1,i_2,\dots,i_s}\to\mathcal{W}_{i_1,i_2,\dots,i_s}$$

$$(z_1,z_2,\dots,z_n,z_{n+1})\mapsto(z_1,\dots,z_{i_1-1},z_{i_1}-k,z_{i_1+1},\dots,z_{i_s-1},z_{i_s}-k,z_{i_s+1},\dots,z_{n+1})$$

which subtracts k on the $i_1,i_2,\dots,i_s$-th elements. Each element in $\mathcal{W}_{i_1,i_2,\dots,i_s}$ has one and only one inverse image, so that $f_{i_1,i_2,\dots,i_s}$ is an one-one correspondence. Since $\mathcal{Z}_{i_1,i_2,\dots,i_s}$ and $\mathcal{W}_{i_1,i_2,\dots,i_s}$ are finite sets, we have

$$\#(\mathcal{Z}_{i_1,i_2,\dots,i_s})=\#(\mathcal{W}_{i_1,i_2,\dots,i_s})=\binom{l-ks}{n}.$$

Let $\binom{l-ks}{n}=0$ when $l-ks\leq 0$, then we have

$$\#\left(\bigcup_{j=1}^{n+1}\{Z_j\geq k\}\right)=\sum_{s=1}^{n+1}(-1)^{s-1}\binom{n+1}{s}\binom{l-ks}{n}.$$

The above formula can be written as

$$\#\left(\bigcup_{j=1}^{n+1}\{Z_j\geq k\}\right)=\sum_{s=1}^{Q(n)}(-1)^{s-1}\binom{n+1}{s}\binom{l-ks}{n},\tag{4}$$

where $Q(n)=\max\{s|l-ks\geq n\}\wedge(n+1)$. Here "$\wedge$" means taking the smaller one between two values. The occasion when $n=0$ is also included in the above formula. The combination of (1), (2), (3) and (4) gives the expression of $\tau(k,m,p,l)$.

According to the definition of M and K, it is easy to know that $\{K\geq k,M\leq m\}\subset\{K\geq k,M\leq m+1\}$ and $\{K\geq k,M\leq m\}\supset\{K\geq k+1,M\leq m\}$ when l and p are fixed. Therefore the first two conclusions hold.

We prove that τ decreases with p below.

Denote E_n $(0\leq n\leq l)$ as a set of l-dimension 0-1 vectors. Each element in E_n contains exactly n 1s, and the maximal length of continuous 0s is equal to or greater than k. According to the above steps,

$$\tau(k,m,p,l)=\sum_{n=0}^{m}\#(E_n)p^n(1-p)^{l-n}.$$

Taking the derivative of τ with respect to p gives

$$\begin{aligned}\frac{\partial\tau(k,m,p,l)}{\partial p} &= \sum_{n=0}^{m}\#(E_n)np^{n-1}(1-p)^{l-n} - \sum_{n=0}^{m}\#(E_n)(l-n)p^n(1-p)^{l-n-1}\\ &= \sum_{n=1}^{m}\#(E_n)np^{n-1}(1-p)^{l-n} - \sum_{n=0}^{m}\#(E_n)(l-n)p^n(1-p)^{l-n-1}\\ &= \sum_{n=0}^{m-1}\#(E_{n+1})(n+1)p^{n}(1-p)^{l-n-1} - \sum_{n=0}^{m}\#(E_n)(l-n)p^n(1-p)^{l-n-1}\\ &= \sum_{n=0}^{m-1}\left[(n+1)\#(E_{n+1})-(l-n)\#(E_n)\right]p^{n}(1-p)^{l-n-1} - (l-m)\#(E_m)p^m(1-p)^{l-m-1}\end{aligned}$$

Denote $\boldsymbol{e}$ as an l-dimension 0-1 vector and e_i as its i-th entry. For any $n(0 \leq n \leq l-1)$, define two sets $\mathscr{B}$ and $\mathscr{B}^{'}$ as:

$$\mathscr{B} = \{(\boldsymbol{e}, i)|\boldsymbol{e} \in E_{n+1}, e_i = 1, 1 \leq i \leq l\}$$
$$\mathscr{B}^{'} = \{(\boldsymbol{e}, i)|\boldsymbol{e} \in E_{n}, e_i = 0, 1 \leq i \leq l\}$$

Let F be the mapping from $\mathscr{B}$ to $\mathscr{B}^{'}$:

$$F : \mathscr{B} \rightarrow \mathscr{B}^{'}$$
$$(\boldsymbol{e}, i) \mapsto (f_i(\boldsymbol{e}), i)$$

f_i is the operator that changes the i-th entry of $\boldsymbol{e}$ as 0. It can be verified that F is a single map, so that

$$\#(\mathscr{B}) \leq \#(\mathscr{B}^{'}).$$

This implies

$$(n+1)\#(B_{n+1}) \leq (l-n)\#(B_n).$$

Therefore $\frac{\partial\tau(k,m,p,l)}{\partial p} \leq 0$ and τ decreases with p.

□

2 Dependence of short read alignment

we study the distribution of N, which is the number of aligned short reads. Given the errors in the long reads, the alignments of all short reads are not completely independent. Once the shift distance of two short reads (i.e., the distance between their start positions) is small and one of them fails to be aligned, it is highly likely that the other cannot be aligned (Fig. S1A) as both short reads cover nearly the same set of errors on long reads. The correlation of mismatch numbers between two short reads decreases with their shift distance, and reaches low value when the shift distance is > 30 bp (Fig. S1B). As the short read length is 100 bp in this study, all short reads covering the base b of interest are divided into three clusters based on their start positions (Fig. S1C). Assume that (1) the start positions of the short reads are uniformly distributed; (2) whether the short reads in different clusters can be successfully aligned is independent; (3) the short reads in each cluster are all aligned or all not aligned. Denote

$\boldsymbol{D} = (D_1, D_2, D_3)$ as the number of sequencing short reads in three clusters, and $\boldsymbol{d} = (d_1, d_2, d_3)$ as the value of $\boldsymbol{D}$. Note that $D_1 + D_2 + D_3 = C$. Let $N_i (1 \le N_i \le 3)$ be the random variable that represents the number of aligned short reads in the i-th cluster. The observed aligned short reads N equals to $N_1 + N_2 + N_3$. The probability $\Pr(N = n)$ can be expressed as

$$\Pr(N = n) = \sum_{\substack{d_1, d_2, d_3 \\ d_1 + d_2 + d_3 = C}} \Pr(N = n | \boldsymbol{D} = \boldsymbol{d}) \Pr(\boldsymbol{D} = \boldsymbol{d})$$

According to assumption (1), $\Pr(\boldsymbol{D} = \boldsymbol{d})$ can be calculated based on the trinomial distribution $\text{Trinom}(C; \frac{1}{3}, \frac{1}{3}, \frac{1}{3})$. According to assumption (2) and (3),

$$\Pr(N = n | \boldsymbol{D} = \boldsymbol{d}) = \sum_{\substack{n_1, n_2, n_3 \\ n_1 + n_2 + n_3 = n, n_i \in \{0, d_i\}}} \Pr(N_1 = n_1) \Pr(N_2 = n_2) \Pr(N_3 = n_3)$$

where $\Pr(N_i = n_i) = \tau$ if $n_i = 0$, and $1 - \tau$ if $n_i = d_i$.

3 Estimation of mismatch rate p

Denote the short read error rate and long read error rate as β and γ, respectively. Let S_i be the real nucleotide on the molecule. Let X_i and Y_i be the corresponding nucleotides on the short read and long read. Since the short read and long read are sequenced independently, we can simply assume that $\{X_i = S_i\}$ and $\{Y_i = S_i\}$ are independent. The mismatch rate is calculated as

$$\begin{aligned}
p =& \Pr(Y_i \neq X_i) \\
=& \Pr(S_i = X_i, Y_i \neq S_i) + \Pr(S_i \neq X_i, Y_i = S_i) + \Pr(S_i \neq X_i, Y_i \neq S_i, Y_i \neq X_i) \\
=& \Pr(S_i = X_i)\Pr(Y_i \neq S_i | S_i = X_i) + \Pr(S_i \neq X_i)\Pr(Y_i = S_i | S_i \neq X_i) \\
&+ \Pr(S_i \neq X_i)\Pr(Y_i \neq S_i | S_i \neq X_i)\Pr(Y_i \neq X_i | Y_i \neq S_i, S_i \neq X_i) \\
=& \Pr(S_i = X_i)\Pr(Y_i \neq S_i) + \Pr(S_i \neq X_i)\Pr(Y_i = S_i) \\
&+ \Pr(S_i \neq X_i)\Pr(Y_i \neq S_i)\Pr(Y_i \neq X_i | Y_i \neq S_i, S_i \neq X_i) \\
=& (1 - \beta)\gamma + \beta(1 - \gamma) + \beta\gamma\Pr(Y_i \neq X_i | Y_i \neq S_i, S_i \neq X_i).
\end{aligned}$$

It can be seen that

$$(1 - \beta)\gamma + \beta(1 - \gamma) \le p \le (1 - \beta)\gamma + \beta(1 - \gamma) + \beta\gamma,$$

which indicates that

$$\beta + \gamma - 2\beta\gamma \le p \le \beta + \gamma + \beta\gamma.$$

Note that γ generally ranges between 5% and 30%, while β is only from 0.1% to 1%. The value of $\beta\gamma$ is usually tiny, so we can simply set the mismatch rate p as $\beta + \gamma$, which is the summation of the short read error rate and the long read error rate. The ranges of γ and β also imply that the mismatch rate p is mainly determined by the long read error rate.

4 Relationship between τ and parameters p, k, m

We further study how τ changes with certain values of p, k, and m (Fig. 1B). l is set as 100, the common length of Illumina short reads. τ shows inverse sigmoidal tendency with respect to p: τ is near 1 when p is small, and τ decreases rapidly to 0 as p increases. This indicates that the mismatch rate, or approximately the long read error rate, is the most dominant factor on τ. The start and end of the dramatic drop depend on k and m. Under the regular settings of k (from 15 to 20) and m (from 10 to 20), the drop occurs as $5\% < p < 30\%$ (Fig. 1B), which is the same as the typical range of TGS long read error rate. Thus, the alignment-based method is applicable to correct TGS data. As m increases, both τ and the effect of k on τ increase. When $m = 10$, the increment of k from 15 to 20 has little effect on τ and the dramatic drop of τ starts at $p = 5\%$ and ends at $p = 20\%$ (Fig. 1B). As m increases to 15 and 20, the effect of k becomes more obvious, and the dramatic drop of τ shifts to higher mismatch rate, starting at $p = 10\%$ and ending at $p = 25\%$ and $p = 30\%$, respectively.

The probability that a short read is aligned to a random place, or an incorrect place, can also be calculated using Theorem 1. The parameter p should be accordingly replaced by the mismatch rate under random occasion, which can be taken as 75%. Under a most loose criterion: $k = 12$, $m = 40$, the probability of successful alignment in random occasion is $\tau(12, 40, 0.75, 100) = 7.63 \times 10^{-15}$. This implies that even if we have 300G Illumina short reads (10,000× coverage for human genome), the expected number of randomly aligned short read at each base is only 2.29×10^{-3}. We therefore neglect the randomly aligned short reads. It should be noticed that by doing so we also neglect the false alignment of the short reads generated from repetitive regions. In that occasion, the mismatch rate p is not fixed and the problem is hard to model.

5 Monotonicity of consensus inference accuracy

$g(N, \beta)$ is the consensus inference accuracy and is defined as (Methods part in main text):

$$g(N,\beta) = \Pr\left(W_{N,1-\beta} > \frac{N-1}{2}\right), \qquad N \text{ is odd}$$

$$g(N,\beta) = \Pr\left(W_{N,1-\beta} > \frac{N}{2}\right) + \frac{1}{2}\Pr\left(W_{N,1-\beta} = \frac{N}{2}\right), \qquad N \text{ is even}$$

$W_{N,\beta}$ follows the binomial distribution $\text{Binom}(N, 1-\beta)$. N is the random variable that represents the number of successfully aligned short read covering the target base, and let n be a specific value of n. β is the short read error rate.

Next, we prove that $g(n, \beta)$ increases with n and decreases with β. The conclusions can be obtained from the following two lemmas.

Lemma 1. *Suppose n is a positive even number, then $g(n, \beta) = g(n-1, \beta)$. If $\beta < \frac{1}{2}$, we also have $g(n, \beta) < g(n+1, \beta)$.*

Proof. We prove the fist conclusion. When n is even, we have

$$\begin{aligned}
g(n,\beta) &= \Pr\left(W_{n,1-\beta} > \frac{n}{2}\right) + \frac{1}{2}\Pr\left(W_{n,1-\beta} = \frac{n}{2}\right) \\
&= (1-\beta)\Pr\left(W_{n-1,1-\beta} > \frac{n}{2}-1\right) + \beta\Pr\left(W_{n-1,1-\beta} > \frac{n}{2}\right) + \frac{1}{2}\Pr\left(W_{n,1-\beta} = \frac{n}{2}\right) \\
&= (1-\beta)\Pr\left(W_{n-1,1-\beta} > \frac{n}{2}-1\right) + \beta\left[\Pr\left(W_{n-1,1-\beta} > \frac{n}{2}-1\right) - \Pr\left(W_{n-1,1-\beta} = \frac{n}{2}\right)\right] \\
&+ \frac{1}{2}\Pr\left(W_{n,1-\beta} = \frac{n}{2}\right) \\
&= \Pr\left(W_{n-1,1-\beta} > \frac{n}{2}-1\right) - \beta\Pr\left(W_{n-1,1-\beta} = \frac{n}{2}\right) + \frac{1}{2}\Pr\left(W_{n,1-\beta} = \frac{n}{2}\right).
\end{aligned}$$

Note that

$$\begin{aligned}
&\frac{1}{2}\Pr\left(W_{n,1-\beta} = \frac{n}{2}\right) - \beta\Pr\left(W_{n-1,1-\beta} = \frac{n}{2}\right) \\
=&\frac{1}{2}(1-\beta)\Pr\left(W_{n-1,1-\beta} = \frac{n}{2}-1\right) + \frac{1}{2}\beta\Pr\left(W_{n-1,1-\beta} = \frac{n}{2}\right) - \beta\Pr\left(W_{n-1,1-\beta} = \frac{n}{2}\right) \\
=&\frac{1}{2}(1-\beta)\Pr\left(W_{n-1,1-\beta} = \frac{n}{2}-1\right) - \frac{1}{2}\beta\Pr\left(W_{n-1,1-\beta} = \frac{n}{2}\right) \\
=&\frac{1}{2}(1-\beta)\binom{n-1}{\frac{n}{2}-1}(1-\beta)^{\frac{n}{2}-1}\beta^{\frac{n}{2}} - \frac{1}{2}\beta\binom{n-1}{\frac{n}{2}}(1-\beta)^{\frac{n}{2}}\beta^{\frac{n}{2}-1} \\
=&\frac{1}{2}\binom{n-1}{\frac{n}{2}}(1-\beta)^{\frac{n}{2}}\beta^{\frac{n}{2}} - \frac{1}{2}\binom{n-1}{\frac{n}{2}}(1-\beta)^{\frac{n}{2}}\beta^{\frac{n}{2}} \\
=&0.
\end{aligned}$$

Therefore

$$g(n,\beta) = \Pr\left(W_{n-1,\beta} > \frac{n}{2}-1\right) = g(n-1,\beta),$$

thus the first conclusion holds.

When $\beta < \frac{1}{2}$,

$$\begin{aligned}
&g(n+1,\beta) - g(n,\beta) \\
=&\Pr\left(W_{n+1,1-\beta} > \frac{n}{2}\right) - \Pr\left(W_{n,1-\beta} > \frac{n}{2}\right) - \frac{1}{2}\Pr\left(W_{n,1-\beta} = \frac{n}{2}\right) \\
=&(1-\beta)\Pr\left(W_{n,1-\beta} > \frac{n}{2}-1\right) + \beta\Pr\left(W_{n,1-\beta} > \frac{n}{2}\right) - \Pr\left(W_{n,1-\beta} > \frac{n}{2}\right) - \frac{1}{2}\Pr\left(W_{n,\theta} = \frac{n}{2}\right) \\
=&(1-\beta)\left[\Pr\left(W_{n,1-\beta} > \frac{n}{2}-1\right) - \Pr\left(W_{n,1-\beta} > \frac{n}{2}\right)\right] - \frac{1}{2}\Pr\left(W_{n,1-\beta} = \frac{n}{2}\right) \\
=&\left(\frac{1}{2}-\beta\right)\Pr\left(W_{n,1-\beta} = \frac{n}{2}\right) \\
>&0,
\end{aligned}$$

therefore the second conclusion holds. Due to the nature of Second Generation Sequencing, $\beta < \frac{1}{2}$ almost holds in any time, so we can generally conclude that $g(n,\beta)$ decreases with short read error rate β. □

Lemma 2. *$g(n,\beta)$ decreases with respect to β.*

Proof. According to Lemma 1, we only need to prove the occasion when n is odd, when

$$g(n,\beta) = \Pr\left(W_{n,1-\beta} > \frac{n-1}{2}\right) = 1 - \sum_{m=0}^{\frac{n-1}{2}} \binom{n}{m} (1-\beta)^m \beta^{n-m}.$$

Taking the derivative of $g(n,\beta)$ with respect to β gives

$$\begin{aligned}
\frac{\partial g(n,\beta)}{\partial \beta} &= \sum_{m=0}^{\frac{n-1}{2}} \binom{n}{m} m(1-\beta)^{m-1}\beta^{n-m} - \sum_{m=0}^{\frac{n-1}{2}} \binom{n}{m}(n-m)(1-\beta)^m\beta^{n-m-1} \\
&= \sum_{m=1}^{\frac{n-1}{2}} \binom{n}{m} m(1-\beta)^{m-1}\beta^{n-m} - \sum_{m=0}^{\frac{n-1}{2}} \binom{n}{m}(n-m)(1-\beta)^m\beta^{n-m-1} \\
&= \sum_{m=0}^{\frac{n-3}{2}} \binom{n}{m+1} (m+1)(1-\beta)^{m}\beta^{n-m-1} - \sum_{m=0}^{\frac{n-1}{2}} \binom{n}{m}(n-m)(1-\beta)^m\beta^{n-m-1} \\
&= \sum_{m=0}^{\frac{n-3}{2}} \left[\binom{n}{m+1}(m+1) - \binom{n}{m}(n-m)\right](1-\beta)^m\beta^{n-m-1} - \binom{n}{\frac{n-1}{2}}\left(\frac{n+1}{2}\right)(1-\beta)^{\frac{n-1}{2}}\beta^{\frac{n-1}{2}} \\
&= -\binom{n}{\frac{n-1}{2}}\left(\frac{n+1}{2}\right)(1-\beta)^{\frac{n-1}{2}}\beta^{\frac{n-1}{2}} \\
&\le 0,
\end{aligned}$$

so that the conclusion holds. □

6 Probability that a k-mer is present in DBG

We calculate p_{DBG}, the probability that a k-mer is present in the DBG, i.e., p_{DBG} equals to the probability that the k-mer is present on at least one short read. Suppose the number of short reads that cover the middle point of the k-mer is C, which is the short read coverage. Assume that the start positions of these short reads are evenly distributed. Let H be the number of short reads that cover the entire k-mer, then H follows the binomial distribution Binom $\left(C, \frac{k}{l}\right)$ and we have

$$\begin{aligned}
p_{DBG} &= \sum_{h=1}^{C} \Pr(H=h)\left(1-\left(1-(1-\beta)^k\right)^h\right) \\
&= \sum_{h=1}^{C} \binom{C}{h}\left(\frac{k}{l}\right)^h\left(1-\frac{k}{l}\right)^{C-h}\left(1-\left(1-(1-\beta)^k\right)^h\right)
\end{aligned}$$

p_{DBG} increases with C and decreases with β and k. We take l as 100 and k as 17, 19 and 21, which are three commonly used k values in software. When the short read error rate is 1%, only 8-9× short

read coverage is required to achieve $p_{DBG} > 99.99\%$. Even if the short read error rate is as high as 2%, the requirement of short read coverage increases to 10-12× for $p_{DBG} > 99.99\%$ (Fig. S3). This shallow coverage is generally satisfied in most current sequencing project. The above analyses imply that the long read length and error rate are the dominant factors for solid k-mer detection.

7 Generation of simulated data

The genome of *E. coli* (strain K-12 MG1655) is taken as the reference to generate simulated data. The long reads are generated using the PacBio read simulator SimLoRD (version 1.0.2) [1]. The parameter `--fixed-readlength` is as 1k, 2k, 5k and 10k, so that reads with four kinds of lengths are generated. Under each of the read length, we set `--probability-threshold` as 1%, 2% and up to 30%, and generate 2,000 long reads for each value. The parameter `--max-passes` for all occasions are set as 0. The software fails to generate any read when the `--probability-threshold` is 1%, so at last 58,000 long reads are generated for each kind of read length. Although SimLoRD automatically outputs the error rate and CIGAR string for each long read, we find that alternate insertion and deletion frequently occur at the places where the long read and reference genome are actually perfectly matched. This implies that the output error rate is likely to be overestimated. We therefore align the long reads to the true fragments on the reference genome and recalculate the long read error rate. The alignment is performed using the Needleman-Wunsch [2] algorithm with the scores of match, mismatch, gap open and gap extension being set as 1, -4, -2 and -1. The long read error rate is calculated as $(\#mismatch + total\_indel\_size)/long\_read\_length$. The notation $\#mismatch$ represents the number of mismatched base pairs. After error rate recalculation, the number of long reads at all error rate levels are not equal to the fixed number 2,000, but they are all greater than 100. This implies that the sample size at each error rate level is still enough to achieve reliable evaluation.

The short reads are generated using ART (version 2.5.8) [3]. The parameter `-ss` (Illumina sequencing system) is set as `HS25`, so that the short reads are simulated with the setting of HiSeq 2500. The short read length is set as 100 bp. We simulate the short reads with three kinds of coverage: 10×, 20× and 50×. We also notice that proovread [4] performs error correction for multiple iterations. In the first iteration, the short reads are split into 1,000 chunks and only 6 chunks in every 20 chunks are used to correct the long reads. It implies that the effective coverage is only 30% of that of the input short reads. In order to mimic the coverage of 5×, 10×, 20× and 50×, we simulate additional short reads with coverage 17×, 33×, 67× and 167× for proovread.

8 Application of proovread

We correct the simulated long reads using proovread (version 5.14.0) [4], a typical and widely-used alignment-based error correction software. Since the model of alignment-based method has no relationship with the length of long read, we arbitrarily select the 2 kb long read set. It should be mentioned that proovread performs iterative correction and different alignment criteria are applied in different rounds. To fairly evaluate the model, we only use the result of the first iteration. In the version 5.14.0, `bwa-proovread`, a modified bwa program is used to align the short reads. The minimum seed length `-k` is automatically set as 12 by proovread. Instead of inputting the whole long reads, we split them

into multiple bins according to their error rates (each bin corresponds to the error rate of 0-3%, 3-6%, 6-9%, 9-12%, 12-15%, 15-18%, 19-21%, 22-24%, 25-27% and 28-30%), and correct them separately. The reason is that when the long reads with high or low quality are mixed together, the short reads tend to be aligned to the high quality ones, and the low quality long reads are probable to embrace no or less short reads. Binning the long reads will reduce this bias and lead to fair evaluation of the model. We also notice that the option `-a` is set in the alignment command, which means all the alignments are output if a short read can be aligned to multiple long reads.

We set $k = 12$ and C as 10, 20 and 50 in the model, which are respectively the seed length threshold and short read coverage. l is set as 100, the short read length. Since proovread does local score comparison rather than applying a single hard cut-off, it is difficult to accurately set a general m. We therefore set m through an approximate approach. Specifically, we calculate the average number of aligned short read at each error rate level for the case of $10\times$ coverage. Then we divide the average number by 200, which is the theoretical maximal aligned short read number, and obtain an alignment rate. We compare the rates at all error rate levels with τ under different m. The value of m minimizes the total difference is selected, which is 20 in our case (Fig. S4). With all parameter being set, the theoretical accuracy gain can be calculated.

For *E. coli* real sequencing data, we also bin the long reads according to their error rates and apply proovread with the default parameters, the same as for the simulated data.

9 Application of LoRDEC

As an evaluation for the model of graph-based method, we use LoRDEC (version 0.5.3) [5], a typical graph-based hybrid error correction software to correct the simulated and real long reads. The common parameter setting is `-a 50000 -s 1`. For simulated data, the k-mer size `-k` is set as values 17, 19 and 21. For *E. coli* real sequencing data, k is set as 19.

10 Processing of real data

We download the PacBio raw data of *E. coli* from PacificBiosciences/DevNet [6]. The bax file is then processed by smrtanalysis (version 2.3.0) with the command `ConsensusTools.sh CircularConsensus --minPredictedAccuracy 75 --minFullPasses 0`. The obtained 55,137 long reads are then aligned to the reference genome of *E. coli* (strain K-12 MG1655) with blasr (version 1.3.1) [7]. The parameters are set as `-bestn 1 -sam -clipping soft`. The number of successful alignments is 53,642. For each of the alignment, we trim the soft-clipped ends of the long read, and take out the region corresponding to the alignment from the reference genome. Those are taken as the pre-corrected and accurate long reads in our tests.

The Illumina short reads are downloaded from NCBI with the accession number ERR022075. In order to mimic $10\times$ short read coverage, we randomly sample $33\times$ (see Note 7 for explanation) and $10\times$ from the short read dataset for proovread [4] and LoRDEC [5], respectively.

The models in the main text rely on the assumption that all bases on a long read are independent and that they share a common error rate. When the models are applied to real data, additional considerations need to be taken. Suppose we have a long read with error rate γ, which implies that the expectation of the total error number (i.e., the sum of mismatch number and total indel size) is $L\gamma$.

We consider two cases: (1) the errors are evenly distributed; (2) errors cluster together. In the latter case, a perfectly matched k-mer is more likely to occur, which implies a greater probability of the long read's being corrected by either the alignment-based or graph-based method. If the frequency of the latter case is substantially larger in real data than under the *i.i.d.* assumption, the accuracy gain could be improperly estimated by the model with the original value of γ, so error rate adjustment is necessary for the analysis of real data.

Given a long read error rate γ, we calculate the distance between every two adjacent errors in the real long reads, and then fit the distance values by a geometric distribution. We pool the values of γ and the rates of the fitted geometric distribution, and then fit a cubic curve $h = h(\gamma)$ over the points (shown in Fig. S6 are examples for the real and simulated *E. coli* datasets). When analyzing the real data, we input $h(\gamma)$ instead of γ to the models. The function h is attributed to the sequencing platform and can be learned using other independent real datasets. We also notice that the error patterns generated by simulation software could be different from real dataset, as the fitted cubic curves do not overlap (Fig. S6).

For the alignment-based method, error correction can only be achieved on the parts of the long reads where short reads can be aligned. At each long read error rate, we calculate the proportion of the regions with short reads being aligned for every long read, and use a boxplot to show the distribution (Fig. S7). The overall distribution of the proportion shows a down-shift tendency when long read error rate increases. Specifically, the entire long reads are likely to be covered by short reads when long read error rate is $< 10\%$. The variance of the distribution increases dramatically after this turning point of long read error rate $\sim 10\%$.

11 Model application to transcriptome sequencing data

In principal, error correction is to correct the error-prone long reads using highly accurate short reads generated from the same resource, in spite of the difference of resource (genome or transcriptome). In addition, long reads are corrected independently. Therefore the algorithms and models mentioned in our study are all applicable to transcriptome sequencing data.

However, two important differences between genome and transcriptome data should be considered: gene expression level and upper limit of transcript length. The unequal expression levels of genes result in uneven short read coverage of transcriptome data, in contrast to the generally uniform distribution of genome sequencing coverage. Therefore, our model based on fixed short read coverage may not work well for this case. Alternatively, one can bin the genes with similar expression levels, and apply our model for every bin, with the tuned short read coverage.

In addition, only a small fraction of transcripts are longer than 10 kb, so in general transcriptome data is relatively shorter than genome data. Using PacBio platform, a large proportion of transcriptome data can generage CCS reads, which is of low error rate ($< 10\%$) and can likely be further corrected "perfectly" by short reads (Fig. 1D and 1F) with both methods. In case of using ONT data, the consensus reads ($1D^2$ or 2D) may still have relatively high error rate. When correcting ONT reads for short transcripts, the alignment-based method may work better (see Fig. 2G).

12 Suggestion on method selection

Long reads are widely used for genome assembly and structural variation detection, while different error correction strategies should be used based on data types and research goals.

When the library with long molecules is sequenced, PacBio likely outputs CLR (continuous long read) data with little self-consensus correction but long length. The long read length can help us to build contigs and scaffolds at the long-range for genome assembly and detect large structural variations. To fully make use of this advantage of CLR data, hybrid error correction can improve the quality of CLR data. Considering the long read length, we suggest the graph-based method as the first choice to correct CLR data as shown in the main text. In addition, the graph-based method is much more computing efficient than the alignment-based method, which is useful to handle the large size of data in genome assembly. Moreover, the long reads failed to be corrected by the graph-based method can be subject to the alignment-based method, and some of them are probably to be rescued.

When short molecules are sequenced, PacBio likely outputs CCS (circular consensus sequencing) data with relatively high accuracy by self-consensus correction but short length. The high accuracy of CCS reads is useful for polishing the sequence accuracy of genome assembly and determining breakpoint of structural variations at single-nucleotide resolution. In some cases, the accuracy of CCS data can reach 99% [8]. In such case, the accuracy is very close to short reads and error correction is just optional.

For ONT data, the consensus reads ($1D^2$ or 2D) may still have relatively high error rate and thus hybrid error correction could still be helpful. For longer ONT reads ($>$ 2 kb), we suggest the graph-based method as the first choice, and to rescue the uncorrected long reads by the alignment-based method. For shorter ONT reads ($<$ 2 kb), the alignment-based method can be taken as the first choice (Fig. 2G), since the solid k-mer in graph-based method is likely to be absent in shorter ONT reads as shown in our analyses.

13 Parameter design for organisms with different genome complexity

One of the most important parameters of error correction methods is the k-mer size, which is used in the alignment-based (threshold for perfectly matched seed length) and the graph-based method (solid k-mer size). Genomes of different organisms can have distinct levels of repetitive sequences, and the parameter k should be specifically designed for each organism to ensure specificity. To do that, we evaluate the specificity by the proportion of unique k-mer pattern count over the genome size (Fig. S8). The specificity increases with k and a turning point is obvious on each curve. The left side of the turning point shows a swift slope, while the right side reaches a plateau of specificity that varies among different organisms. Although a smaller k can lead to higher probability of short read alignment or solid k-mer detection (τ and ϕ in Theorem 1 and its expanded application to graph-based method), it should be controlled by a threshold to reduce false positive rate. Based on the turning point, we suggest $k = 14$ for *E. coli*, $k = 15$ for *S. cerevisiae*, $k = 17$ for *D. melanogaster*, $k = 19$ for *M. musculus* and *H. sapiens* (Fig. S8).

References

[1] Bianca K Stöcker, Johannes Köster, and Sven Rahmann. Simlord: Simulation of long read data. *Bioinformatics*, 32(17):2704–2706, 2016.

[2] Saul B Needleman and Christian D Wunsch. A general method applicable to the search for similarities in the amino acid sequence of two proteins. *Journal of molecular biology*, 48(3):443–453, 1970.

[3] Weichun Huang, Leping Li, Jason R Myers, and Gabor T Marth. Art: a next-generation sequencing read simulator. *Bioinformatics*, 28(4):593–594, 2011.

[4] Thomas Hackl, Rainer Hedrich, Jörg Schultz, and Frank Förster. proovread: large-scale high-accuracy pacbio correction through iterative short read consensus. *Bioinformatics*, 30(21):3004–3011, 2014.

[5] Leena Salmela and Eric Rivals. Lordec: accurate and efficient long read error correction. *Bioinformatics*, 30(24):3506–3514, 2014.

[6] Casey Bergman. Pacific biosciences devnet. *https://github.com/PacificBiosciences/DevNet/wiki/E.-coli-Bacterial-Assembly*, 2017.

[7] Mark J Chaisson and Glenn Tesler. Mapping single molecule sequencing reads using basic local alignment with successive refinement (blasr): application and theory. *BMC bioinformatics*, 13(1):238, 2012.

[8] Aaron M Wenger, Paul Peluso, William J Rowell, Pi-Chuan Chang, Richard J Hall, Gregory T Concepcion, Jana Ebler, Arkarachai Fungtammasan, Alexey Kolesnikov, Nathan D Olson, et al. Accurate circular consensus long-read sequencing improves variant detection and assembly of a human genome. *Nature biotechnology*, pages 1–8, 2019.

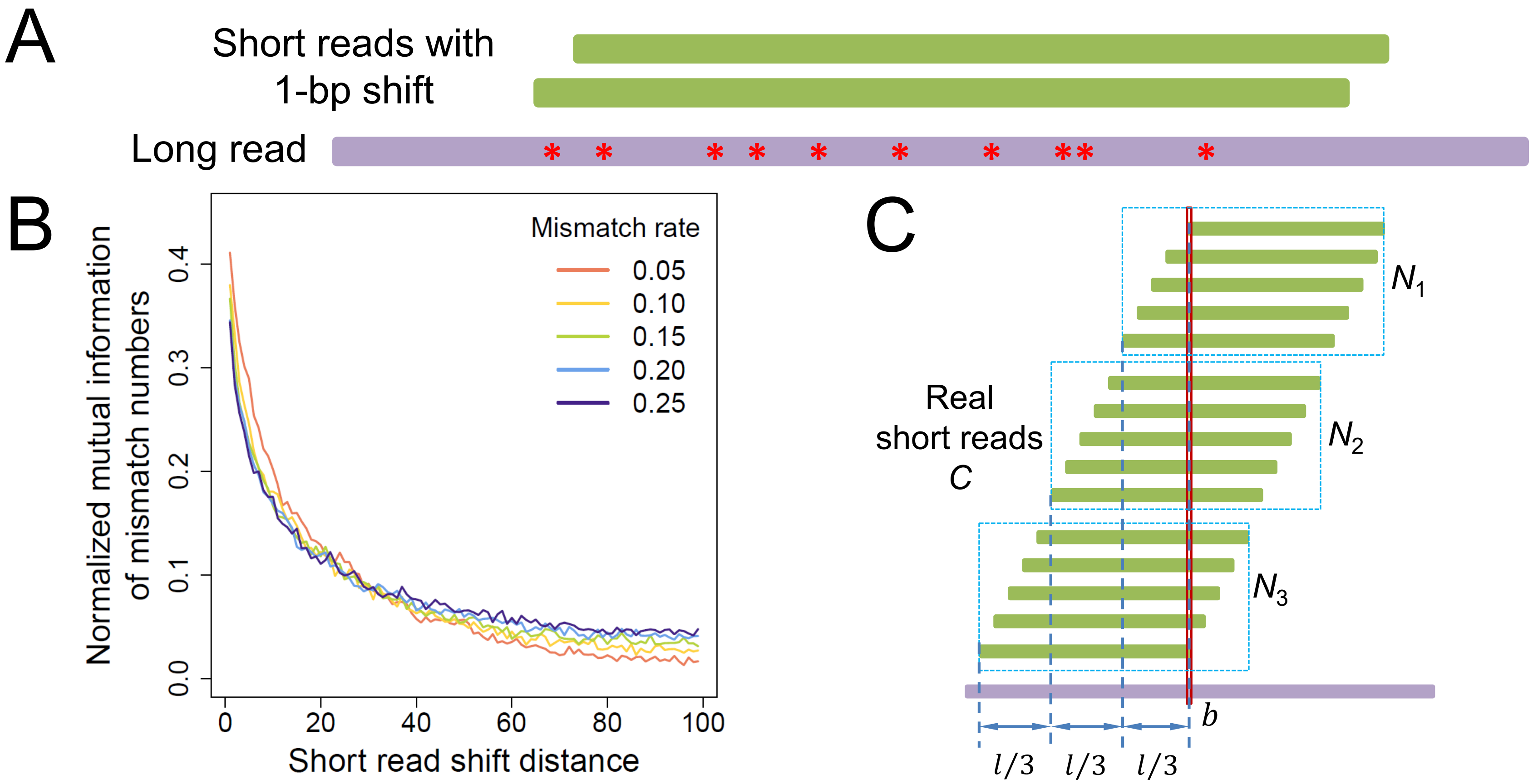

Fig. S1: Independence of short read alignment and the related model. (A) A case showing the dependency of short read alignments. The lower short read has 10 mismatches (represented by red stars) with the long read. The upper short read has only 1 bp shift with the lower one, so that the difference of mismatch numbers for the two short reads cannot exceed 1. Under a certain alignment criterion, if one of the short read can (cannot) be aligned, then it is high likely that the other can (cannot) be aligned. Therefore it is not proper to assume that whether all short reads can be successfully aligned is independent. **(B)** Relationship between the NMI (normalized mutual information) of mismatch numbers and short read shift distance revealed by simulated tests. The correlation of mismatch number decreases when the shift distance increases. **(C)** Proposed model to tackle short read alignment dependence. The real short reads covering a certain base are divided into three classes according to their positions. The numbers of short reads in the three classes are denoted as N_1, N_2 and N_3. It is assumed that the alignments of the short reads in different classes are independent, while all short reads within a class can (cannot) be aligned if one of them is (not) aligned.

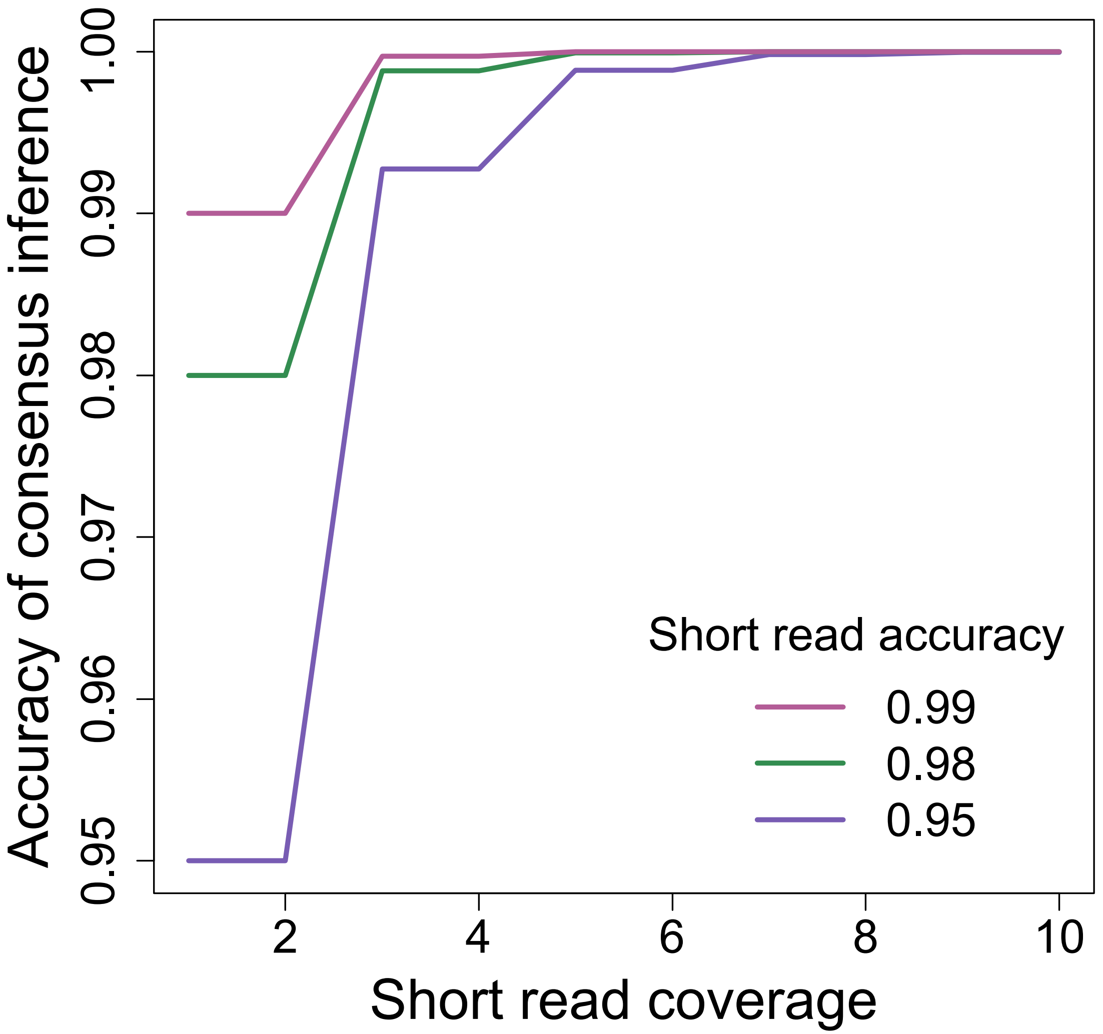

Fig. S2: Relationship between consensus accuracy and short read coverage. The consensus accuracy refers to the probability that the consensus inferred at a base on the long read is equal to the true base. The short read coverage here is the number of aligned short reads that cover the base on the long read. Different curves represent different short read error rates.

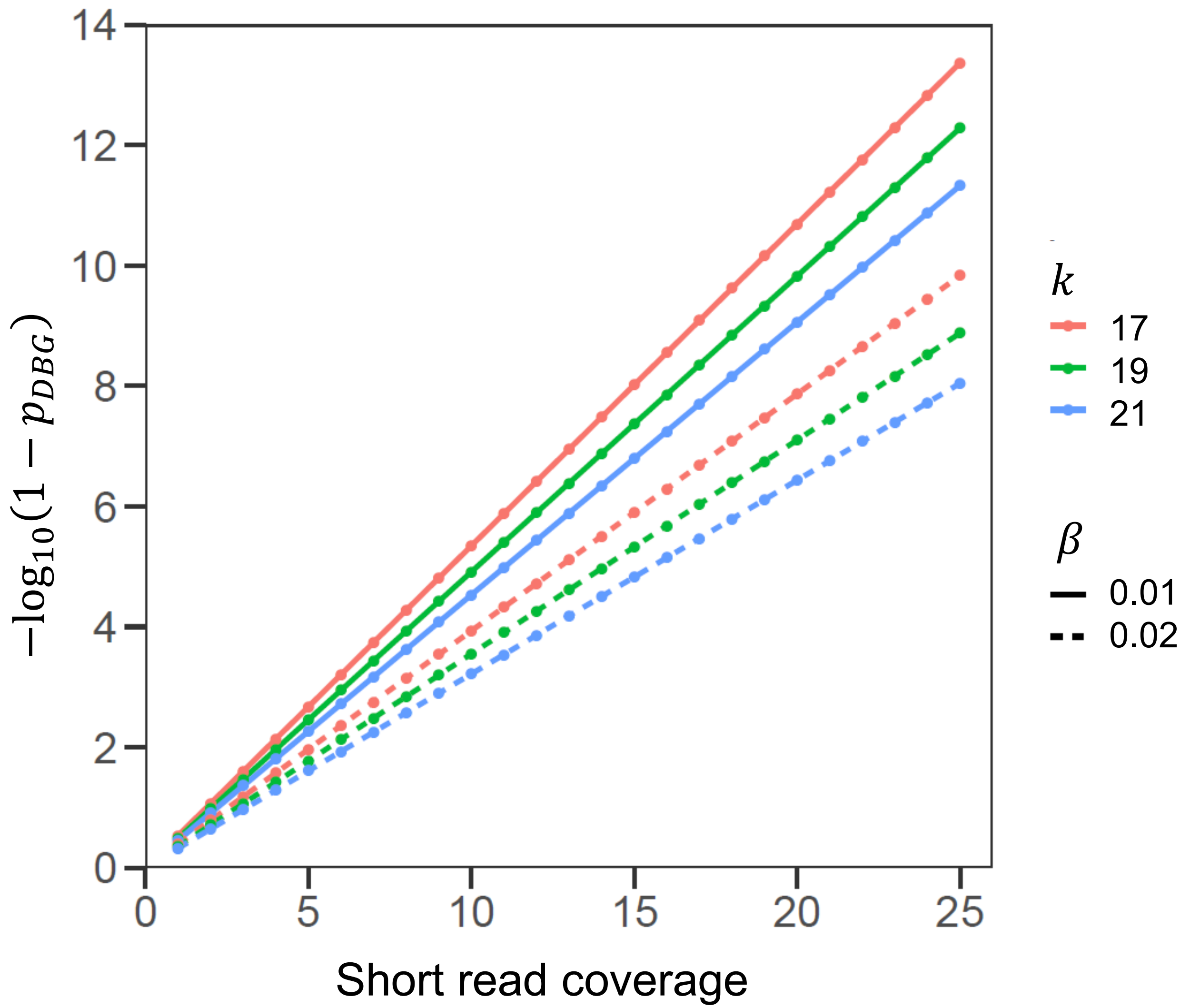

Fig. S3: Relationship between short read coverage and the probability that a solid *k*-mer is present in the DBG created by short reads. The probability is denoted as p_{DBG}, which is transformed by $-\log_{10}(1 - p_{DBG})$. k and β represent the solid *k*-mer size and short read error rate, respectively.

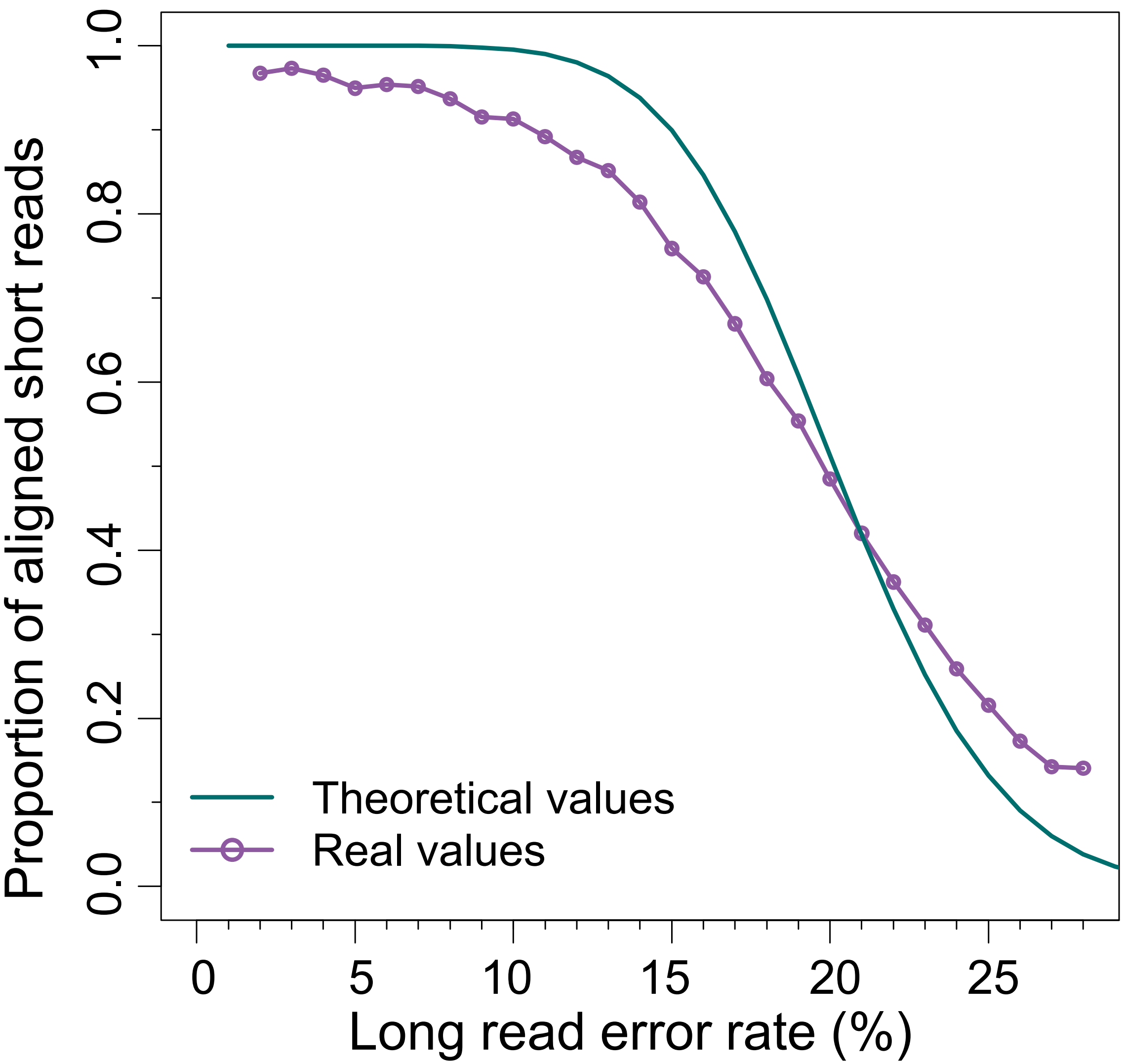

Fig. S4: Selection of mismatch threshold m for the model of alignment-based method. Based on the short read alignment results (10× coverage) in proovread for simulated data, we calculate the average number of aligned short reads on each long read for different error rate levels. The average numbers are then divided by 200, which is the theoretical maximal number of aligned short reads. The obtained rates are shown by the purple curve. For different m, we subtract the obtained rate by $\tau(12, m, p, 100)$ at each error rate, and calculate the sum of the absolute values. The m that minimizes the summation is selected and is used in the test of model fitness. In our case, m is selected as 20 and the corresponding theoretical values are shown by the green curve.

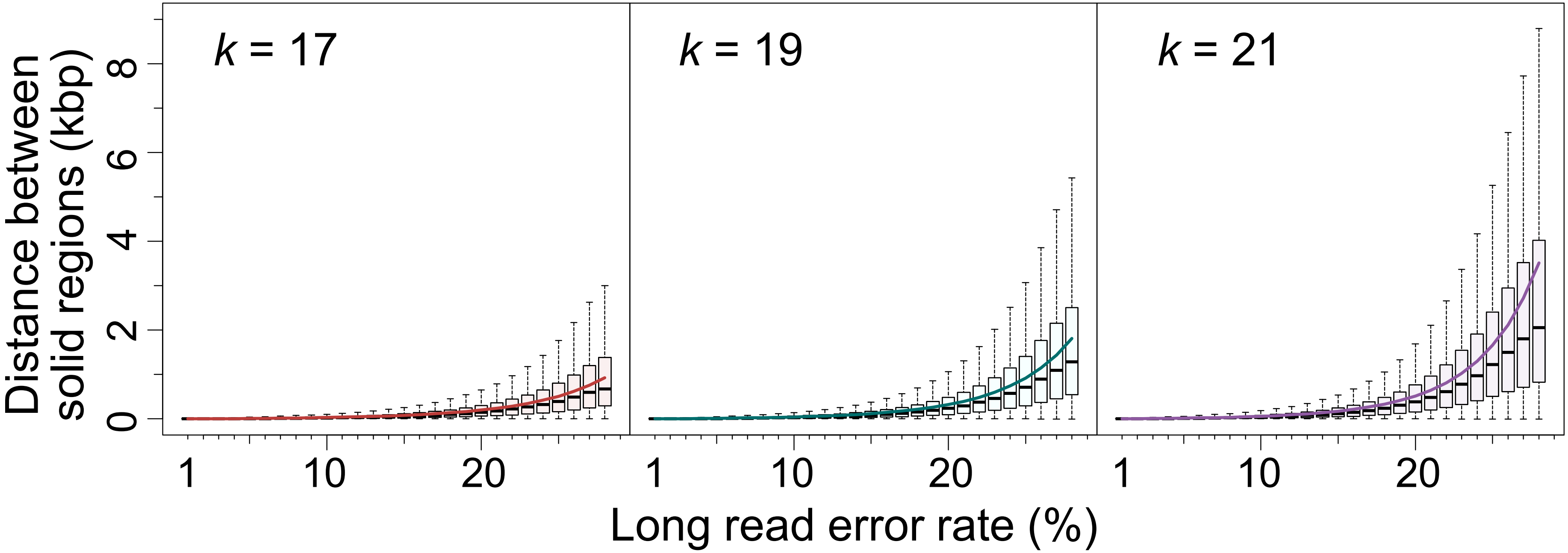

Fig. S5: Relationship between adjacent solid region distance and long read error rate. The boxplots represent the solid region distances on the simulated long reads. The solid lines represent the expected distance calculated through the model. PacBio and ONT long reads generally contain several thousands of bases, which is in the same order of magnitude as the *y*-axis. k: solid *k*-mer size.

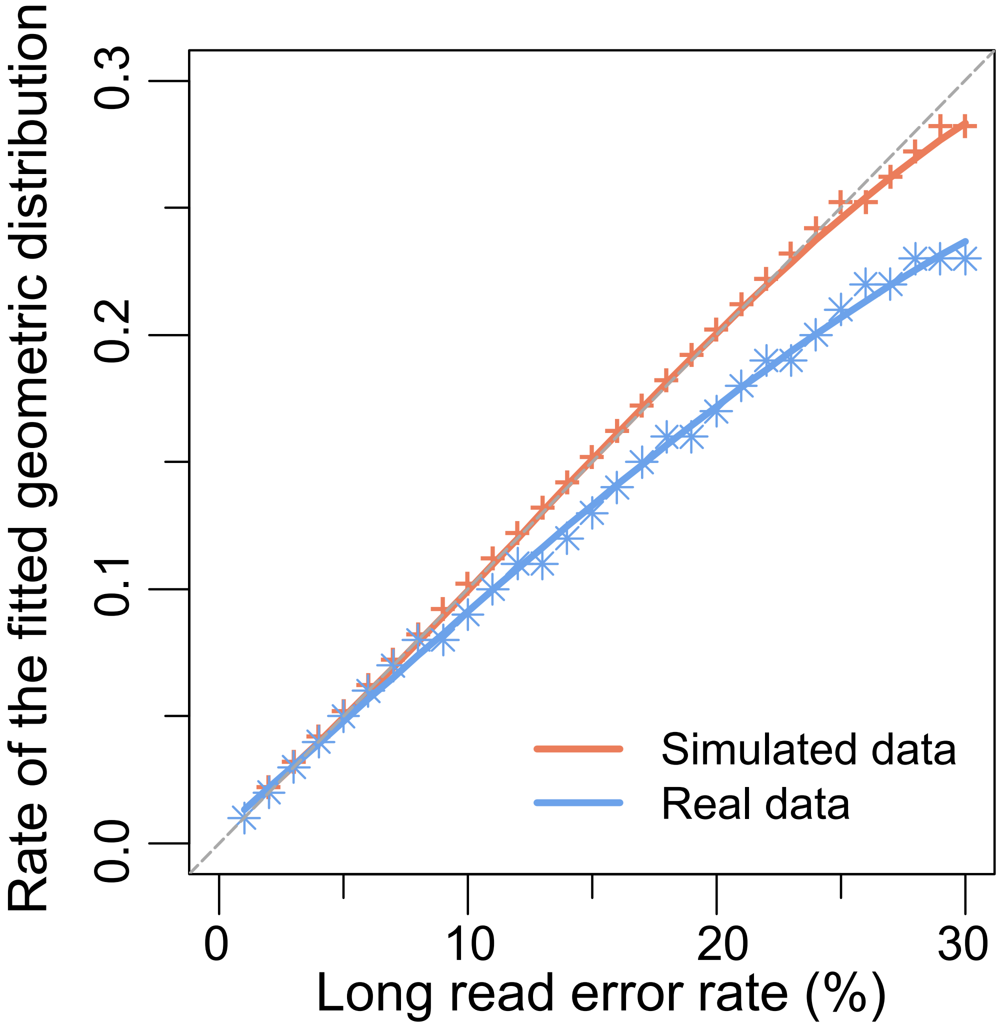

Fig. S6: Relationship between long read error rate and theoretical rate of the fitted geometric distribution. Long reads at each error rate level (represented by *x*-axis) are collected. The distances between every two adjacent errors are calculated and then fitted by a geometric distribution, whose parameter is represented by *y*-axis. The spots are then fitted by two cubic functions, which are shown as the two solid lines.

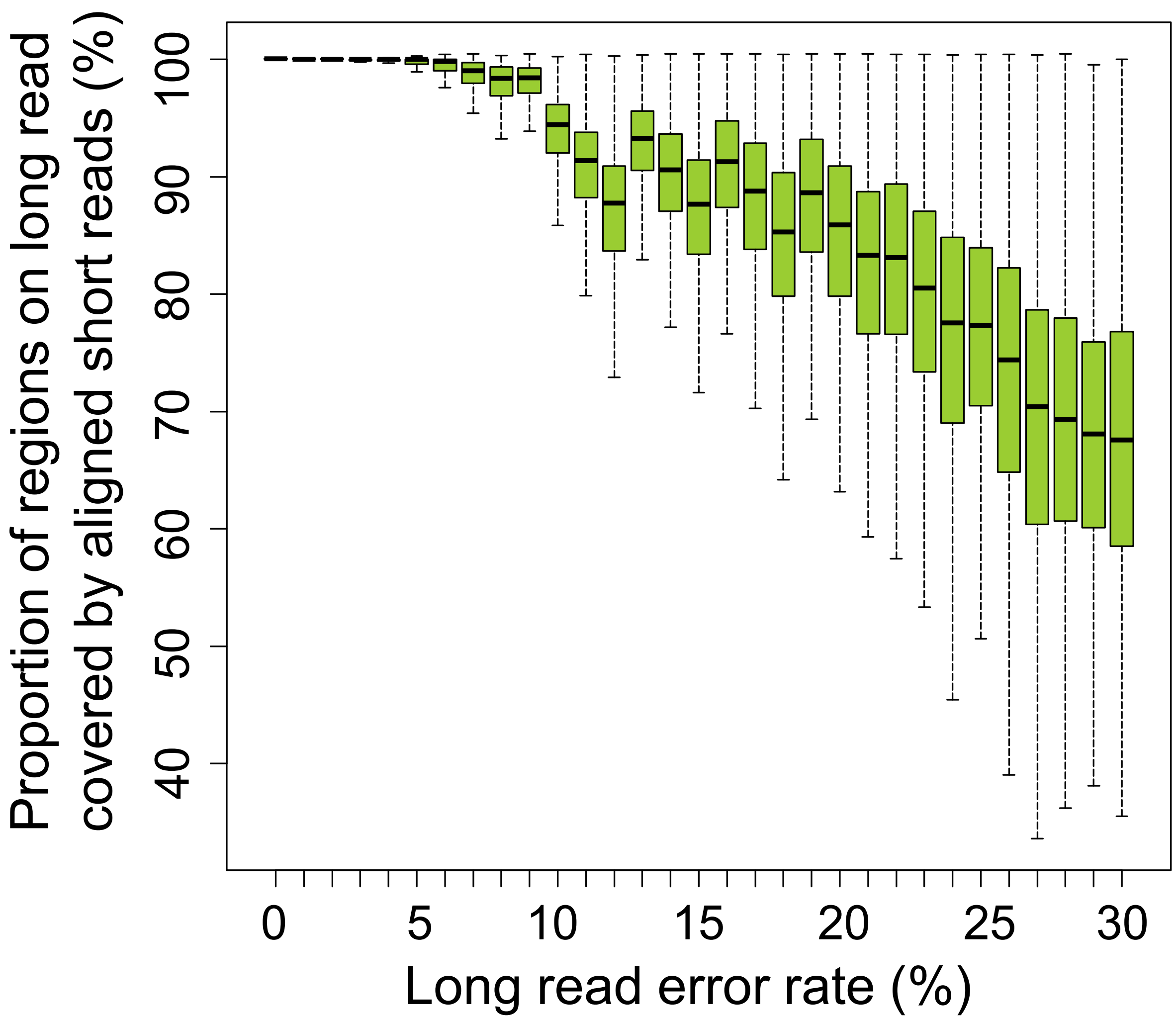

Fig. S7: Relationship between long read error rate and proportion on long read with short reads being aligned. Long reads at each error rate level (represented by *x*-axis) are collected. The proportion of the regions on each long read that are covered by aligned short reads is calculated. Each boxplot represents the distribution of proportion at each long read error rate.

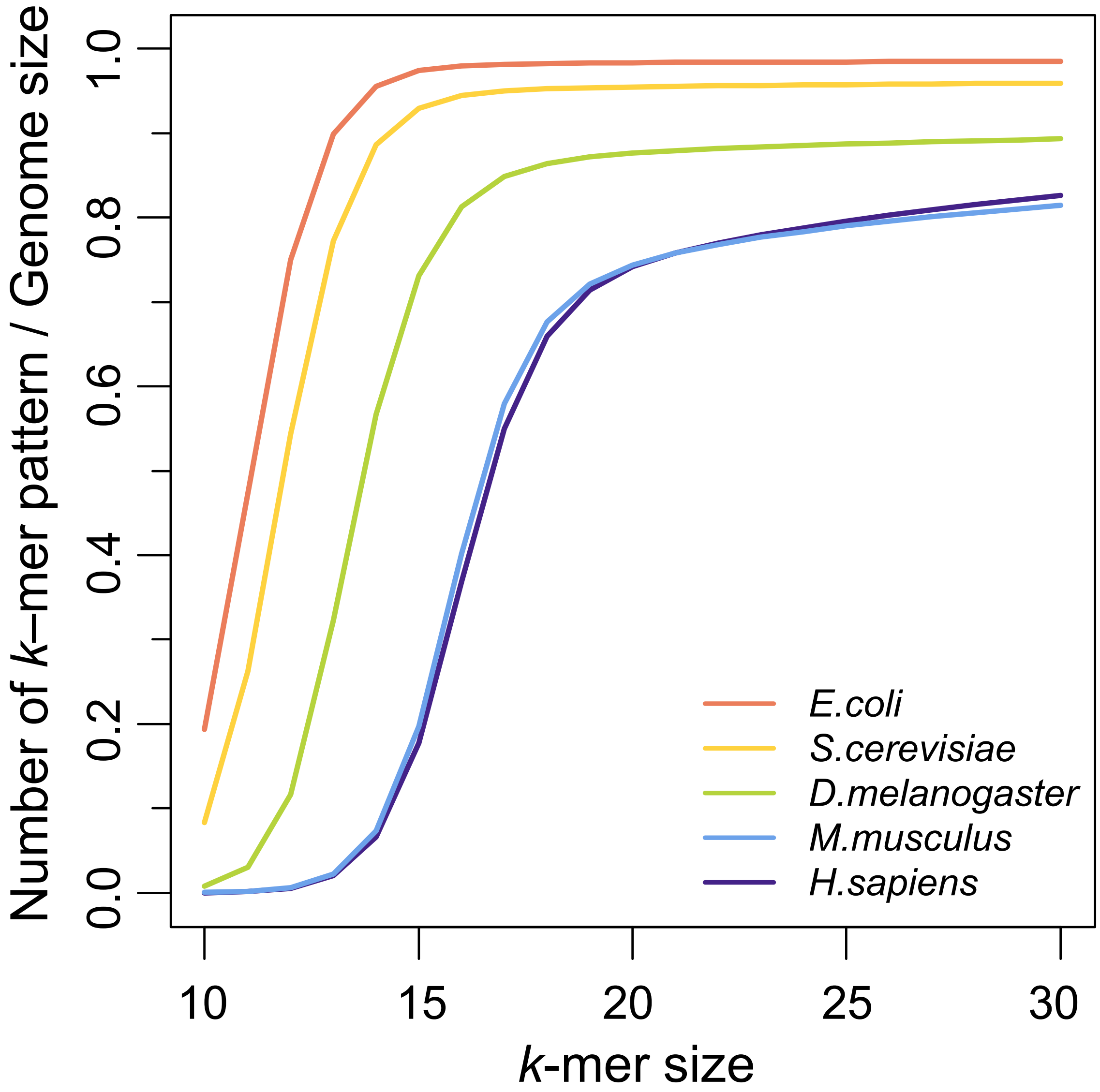

Fig. S8: *k*-mer specificity of the genomes from different organisms. The *k*-mer specificity is measured by the proportion of unique *k*-mer pattern number over the total genome size.
