# Supplementary material for: Performance difference of graph-based and alignment-based hybrid error correction methods for error-prone long reads
Source: Genome Biol. 2020 Jan 17;21:14. doi: 10.1186/s13059-019-1885-y (PMC6966875; doi:10.1186/s13059-019-1885-y)
Supplement: Supplementary file 2 — Additional file 2. Review history. [file 13059_2019_1885_MOESM2_ESM.docx]

Review History

**First round of review**

**Reviewer 1**

**Comments to author:**

Currently, multiple tools have been published to correct the error-prone PacBio /ONT long reads with short reads (“hybrid error correction”). In this manuscript, Wang et al developed a new model, with parameters of long/short read error rate, short read coverage and alignment criterion, to evaluate the performance of two published hybrid error correction tools. With the new model, the authors get some interesting, but not unexpected conclusions on the influences of these parameters. While these conclusions are largely reasonable, I am not fully convinced that they could “offer an informative guidance for method selection, parameter design and future method development for long read error correction”, as the authors claimed.

- Besides the “accuracy gain”, the authors should also evaluate the sensitivity, specificity, and features of corrected reads of these tools with gold standard datasets (such as PacBio self-corrected reads): in many cases, it is more important to adequately control for false positives in reads correction, and get longer corrected reads/contigs, especially when using the strategy of hybrid error correction.

- The authors should clearly define the applications of their conclusions in method selection or parameter design (e.g., genome assembly, identification of structure variations, comparative genomics…etc), and put the findings into the context of the current TGS applications. For example, in genome assembly, long PacBio reads are generated in either CCS or CLR mode, with an error rate less than 15%. For CLR mode, the mean size of reads could reach >10k, which was typically used to generate consensus sequences based on the overlapped regions of long reads. Shorter reads of 1~2k are typically discarded due to limited information; For CCS, although the insert size is much shorter, the reads could be further subject to self-correction with high accuracy. In this case, the major conclusions of this manuscript, such as “the original error rate 19% is the limit for correction”, “the alignment-based method is better for 1~2k raw reads”, and “shallow aligned short read coverage is enough to generate high-accuracy consensus”, are actually not informative for method selection or parameter design.

- It is not safe to simply generalize the optimized parameters in E. coli to other genomes with more complicated sequence structures, such as the human genome with widespread repeat elements: the influences of these tools/parameters in reads correction may change dramatically.

Actually, the authors have published a more comprehensive work on Genome Biology several months ago (Fu et al, Genome Biology, 2019 20:26), to evaluate the performance of ten available error correction methods for long reads, with a similar goal to provide a guideline for the selection of hybrid error correction tools/parameters. That paper is more comprehensive, and adequately addressed some of the issues I mentioned above, such as the specificity, output rate, alignment rate, output read length of these tools. In contrast, this work is a supplement to that study, with relatively limited novelty.

Minor Points

- To highlight the major conclusions of this study, details of the mathematical model in the main text, and some technical details in Fig. 1 and 2 should be moved to supplementary files.
- The order of panels in Fig. 1 should be revised.
- Line 121, “the hybrid error correction benefit marginally from increase of short read coverage, especially when it is greater than 10x”. The panels in Fig.1D looks identical. I cannot directly obtain the conclusion from that figure.

**Reviewer 2**

**Comments to author:**

The manuscript attempts to address a very important topic in effectively using long reads sequencing in biomedical research. It provides a much needed guideline in terms of experimental design on how to bundle long reads sequencing with short reads sequencing. Although many algorithms are available to correct the error-prone long reads with more accurate short read platforms, the overall performance of various methods and their relationship with parameter selection is not fully explored.

The study provided the theoretical calculation on the performance gain with rigorous statistical derivation. The accuracy of the model is further validated using both simulated data and real biological datasets.

Overall, the manuscript is well written, the study addressed an important biological question, and the results are convincing. I do have some minor comments that require some clarification:

1. The errors presented in the long read sequencing are not random, and it is quite different from regular short read platforms. For instance, Nanopore sequencers are notoriously problematic on homopolymers regions, but Illumina sequencing does not have such problem. I would like to see more discussion from this front. I am not sure such bias can be built into the statistical model. However, having some discussion for this will be beneficial.
2. It will be desirable if the authors can provide a little more introduction on the procedure to map the short reads on the long reads. Will the procedure goes through individual long reads independently? Or the correction will be on the long reads consensus? If it is the former, how to address the issue that each short reads can be aligned on multiple long reads.
3. If my understanding is correct, error correction can only be achieve for the portion of the long reads where short reads can be aligned. To be specific, that region needs to have perfect k-mer in the short-reads span to be aligned. It is important to demonstrate, in the real data, what are the percentage of regions the correction can be achieved.
4. It will be interesting to discuss whether similar performance can be achieved for Transcriptome sequencing.

**Authors Response**

**Point-by-point responses to the reviewers’ comments:**

Comment

*Response*

**Reviewer #1:**

Currently, multiple tools have been published to correct the error-prone PacBio /ONT long reads with short reads (“hybrid error correction”). In this manuscript, Wang et al developed a new model, with parameters of long/short read error rate, short read coverage and alignment criterion, to evaluate the performance of two published hybrid error correction tools. With the new model, the authors get some interesting, but not unexpected conclusions on the influences of these parameters. While these conclusions are largely reasonable, I am not fully convinced that they could “offer an informative guidance for method selection, parameter design and future method development for long read error correction”, as the authors claimed.

*Response:*

*We highly appreciate the reviewer’s comments that are very helpful for us. As suggested by the reviewer, we include new discussions about the method selection and parameter design in this revised version. In Additional file 1: Note 13, we provide suggestions on how to choose error correction method at different application scenarios, including genome assembly and structural variation detection. In Additional file 1: Note 14, we provide a strategy on the selection of k-mer size, in which the complexity of a target genome is taken into account.*

*Since this manuscript is for the “Short Report” publication and we have reached the word limit, we add these new contents to Additional file 1 as suggested by the editor. Thank you very much for your understanding.*

- Besides the “accuracy gain”, the authors should also evaluate the sensitivity, specificity, and features of corrected reads of these tools with gold standard datasets (such as PacBio self-corrected reads): in many cases, it is more important to adequately control for false positives in reads correction, and get longer corrected reads/contigs, especially when using the strategy of hybrid error correction.

*Response:*

*We thank the reviewer for the comment. Other than to measure specific software performance metrics of real tools with a few selected parameter settings, the main goal of this work is to theoretically investigate the influences of the principal algorithmic factors on hybrid error correction performance by mathematical modelling. The editor agrees that the manuscript is for a publication of “Short Report” rather than a comprehensive benchmark, and there is no need or space to expand the work completely. Therefore we keep the main contents and “Short Report” style in this resubmission. Please accept our sincere apology and we highly appreciate your understanding.*

- The authors should clearly define the applications of their conclusions in method selection or parameter design (e.g., genome assembly, identification of structure variations, comparative genomics...etc), and put the findings into the context of the current TGS applications. For example, in genome assembly, long PacBio reads are generated in either CCS or CLR mode, with an error rate less than 15%. For CLR mode, the mean size of reads could reach >10k, which was typically used to generate consensus sequences based on the overlapped regions of long reads. Shorter reads of 1~2k are typically discarded due to limited information; For CCS, although the insert size is much shorter, the reads could be further subject to self-correction with high accuracy. In this case, the major conclusions of this manuscript, such as “the original error rate 19% is the limit for correction”, “the alignment-based method is better for 1~2k raw reads”, and “shallow aligned short read coverage is enough to generate high-accuracy consensus”, are actually not informative for method selection or parameter design.

*Response:*

*We thank the reviewer for the suggestion and examples.*

*When the library with long molecules is sequenced, PacBio likely outputs CLR data with little consensus correction but long length. The long read length can help us to build contigs and scaffolds at the long-range for genome assembly and detect large structural variations. To fully make use of this advantage of CLR data, hybrid error correction can improve the quality of CLR data. Considering the long read length, we suggest the graph-based method as the first choice to correct CLR data as shown in the main text. In addition, the graph-based method is much more computing efficient than the alignment-based method, which is useful to handle the large size of data in genome assembly. Moreover, the long reads failed to be corrected by the graph-based method can be subject to the alignment-based method, and some of them are probably to be rescued.*

*When short molecules are sequenced, PacBio likely outputs CCS data with relatively high accuracy by consensus correction but short length. The high accuracy of CCS reads is useful for polishing the sequence accuracy of genome assembly and determining breakpoint of structural variations at single-nucleotide resolution. In some cases, the accuracy of CCS data can reach 99%1. In such case, the accuracy is very close to short reads and error correction is just optional.*

*For ONT data, the consensus reads (1D2 or 2D) may still have relatively high error rate and thus hybrid error correction could still be helpful. For longer ONT reads (> 2 kb), we suggest the graph-based method as the first choice, and to rescue the uncorrected long reads by the alignment-based method. For shorter ONT reads (< 2 kb), the alignment-based method can be taken as the first choice (Fig. 2G), since the solid k-mer in the graph-based method is likely to be absent at short ONT reads as shown in our analyses.*

*We add the above discussion as Additional file 1: Note 13.*

- It is not safe to simply generalize the optimized parameters in E. coli to other genomes with more complicated sequence structures, such as the human genome with widespread repeat elements: the influences of these tools/parameters in reads correction may change dramatically.

*Response:*

*We thank the reviewer for pointing out this issue. In this revision, we provide some suggestions on the selection of k-mer size in different organisms, which is one of the most important*

*parameters in both alignment-based and graph-based methods. Genomes of different organisms can have distinct levels of repetitive sequences, and the parameter k should be specifically designed for each organism to ensure specificity. To do that, we evaluate the specificity by the proportion of unique k-mer pattern count over the genome size (Fig. S8). The specificity increases with k and a turning point is obvious on each curve. The left side of the turning point shows a swift slope, while the right side reaches a plateau of specificity that varies among different organisms. Although a smaller k can lead to higher probability of short read alignment and solid k-mer detection (please see Theorem 1 and its application to graph-based method), it should be controlled by a threshold to reduce false positive rate. Base on the turning point, we suggest = 14 for E. coli, = 15 for S. cerevisiae, = 17 for D. melanogaster, = 19 for M. musculus and H. sapiens (Fig. S8).*

*We add the above discussion to Additional file 1: Note 14.*

Actually, the authors have published a more comprehensive work on Genome Biology several months ago (Fu et al, Genome Biology, 2019 20:26), to evaluate the performance of ten available error correction methods for long reads, with a similar goal to provide a guideline for the selection of hybrid error correction tools/parameters. That paper is more comprehensive, and adequately addressed some of the issues I mentioned above, such as the specificity, output rate, alignment rate, output read length of these tools. In contrast, this work is a supplement to that study, with relatively limited novelty.

*Response:*

*Thank you for your positive comments on our previous work. Other than comparing performance metrics of multiple tools with selected parameter values, we here establish a mathematical model to evaluate the quantitative and continuous influences of different algorithmic factors. For example, it is widely known that the sensitivity of alignment and the following error correction “increases” with smaller k-mer size, but our work provides a mathematical conclusion to measure this negative correlation quantitatively with respect to continuous range of the parameter values. This quantitative measurement will be helpful to optimize the performance in the future method development.*

*We offer our gratitude to the reviewer for understanding our concern and the design of the study.*

Minor Points

- To highlight the major conclusions of this study, details of the mathematical model in the main text, and some technical details in Fig. 1 and 2 should be moved to supplementary files.

*Response:*

*We thank the reviewer for the suggestion on making the manuscript more concise. To highlight the major conclusions, we move the previous Fig. 1F (Relationship between adjacent solid region distance and long read error rate) to Additional file 1: Fig. S5. Since our study is to provide theoretical and quantitative study of error correction algorithms, the Theorem 1 is the kernel result for mathematical modelling. Therefore, we put Theorem 1 and its application to the graph-based method in the main text. All other mathematical details are included in Additional file 1.*

- The order of panels in Fig. 1 should be revised.
*Response:*

*We thank the reviewer for the suggestion on improving the clearness of the figure. We move the previous Fig. 1F to Additional file 1: Fig. S5, and reorder the other panels in this revised version.*

- Line 121, "the hybrid error correction benefit marginally from increase of short read coverage, especially when it is greater than 10x". The panels in Fig.1D looks identical. I cannot directly obtain the conclusion from that figure.

*Response:*

*We thank the reviewer for pointing out this issue. The comment helps us elucidate our argument better. In fact, our conclusion of this part is that the curves are highly similar (though not identical). i.e., "benefit marginally". To address this concern, we perform the calculation below:*

*Let z(y, C) be the median of accuracy gain with respect to long read error rate y and short read coverage C. As C increases from 5, 10, 20 and 50, we notice that max (z(y, 10) - z(y, 5)) =r*

| *0.0194, max (z(y, 20) - z(y, 10)) = 0.0093 and max*  *r r* | *(z(y, 50) - z(y, 20)) = 0.0053. Therefore, the* |
| --- | --- |

*change on the overall accuracy gain is tiny, especially when the short read coverage exceeds 10.*

*We add these numbers to Line 118-119, Page 6.*

Reviewer #2:

The manuscript attempts to address a very important topic in effectively using long reads sequencing in biomedical research. It provides a much needed guideline in terms of experimental design on how to bundle long reads sequencing with short reads sequencing. Although many algorithms are available to correct the error-prone long reads with more accurate short read platforms, the overall performance of various methods and their relationship with parameter selection is not fully explored.

The study provided the theoretical calculation on the performance gain with rigorous statistical derivation. The accuracy of the model is further validated using both simulated data and real biological datasets.

Overall, the manuscript is well written, the study addressed an important biological question, and the results are convincing. I do have some minor comments that require some clarification:

*Response:*

*We thank the reviewer for the positive comments on our work. Based the reviewer’s*

*suggestions below, we mainly calculate the corrected proportions of the real long reads by the aligned short reads, as well as add the discussion of correction of transcriptome data and output of all alignment hits of short reads.*

1. The errors presented in the long read sequencing are not random, and it is quite different from regular short read platforms. For instance, Nanopore sequencers are notoriously problematic on homopolymers regions, but Illumina sequencing does not have such problem. I would like to see more discussion from this front. I am not sure such bias can be built into the statistical model. However, having some discussion for this will be beneficial.

*Response:*

*We thank the reviewer for such a great suggestion. We include discussions of this issue in the manuscript and we may perform detailed investigation in the future research.*

*As the reviewer pointed out, we did notice that the errors in the real long reads were likely to gather into clusters (like the indels) rather than random distribution. Given a long read error rate, the clustering of errors will make more space for the occurrence of perfectly matched k-mer for the alignment-based or solid k-mer for the graph-based method. Thus, the correction of the real data should be better than the theoretical calculation based on the random distribution of long-read error.*

*We therefore adjust the long read error rate before its being input to the model to achieve more accurate prediction. Specifically, we select the long reads with a certain error rate, calculate the distance between adjacent errors, and fit a geometric distribution. We then fit a function*

*between the real error rates and the obtained parameters of geometric distribution, and the function can serve as the adjustment of long read error rate.*

*We add a discussion in the main text, Line 201-204 and more details can be found in Additional file 1: Note 11 and Fig. S6.*

2.It will be desirable if the authors can provide a little more introduction on the procedure to map the short reads on the long reads. Will the procedure goes through individual long reads independently? Or the correction will be on the long reads consensus? If it is the former, how to address the issue that each short reads can be aligned on multiple long reads.

*Response:*

*We thank the reviewer for the questions. Instead of generating long read consensus, the alignment-based method aligns the short reads to individual long reads independently. The alignment-based software “proovread” used in this study adopts BWA to map the short reads. It sets the option “-a” in running BWA, in which all the alignment hits are output.*

*We add this remark at the end of the first paragraph in Additional file 1: Note 9.*

3.If my understanding is correct, error correction can only be achieve for the portion of the long reads where short reads can be aligned. To be specific, that region needs to have perfect k-mer in the short-reads span to be aligned. It is important to demonstrate, in the real data, what are the percentage of regions the correction can be achieved.

*Response:*

*We thank the reviewer for this insightful suggestion. It is indeed an important aspect to study. We calculate the percentage of regions that the error correction can be achieved for the real long reads and the new figure Fig. S7 is added. The overall distribution of the corrected percentage shows a down-shift tendency when long read error rate increases.*

*We add the discussion in the last paragraph in Additional file 1: Note 11.*

4. It will be interesting to discuss whether similar performance can be achieved for Transcriptome sequencing.

*Response:*

*We thank the reviewer for pointing out this issue. Indeed, error correction in transcriptome data is also very important, such as for accurate splice detection.*

*In principal, error correction is to correct the error-prone long reads using highly accurate short reads generated from the same resource, in spite of the difference of resource (genome or transcriptome). In addition, long reads are corrected independently. Therefore the algorithms and models mentioned in our study are all applicable to transcriptome sequencing data.*

*However, two important differences between genome and transcriptome data should be considered: gene expression level and upper limit of transcript length. 1) The unequal expression levels of genes result in uneven short read coverage of transcriptome data, in contrast to the generally uniform distribution of genome sequencing coverage. The highly expressed genes have higher short read coverage than that of lowly expressed genes. Therefore, our model based on fixed short read coverage may not work well for this case. Alternatively, one can bin the genes with similar expression levels, and apply our model for every bin, with the tuned short read coverage. 2) Only a small fraction of transcripts are longer than 10 kb, so in general transcriptome data is relatively shorter than genome data. Using PacBio data, a large proportion of transcriptome data can generate CCS reads, which is of low error rate (< 10%) and can likely be further corrected “perfectly” by short reads (Fig. 1D and 1F) with both methods. In case of using ONT data, the consensus reads (1D2 or 2D) may still have relatively high error rate. When correcting ONT reads for short transcripts, the alignment-based methods may work better (please see Fig. 2G)*

*We add the discussion in the main text, Line 201-204 and more details can be found in Additional file 1: Note 12.*

*Reference: Aaron M Wenger, Paul Peluso, William J Rowell, Pi-Chuan Chang, Richard J Hall, Gregory T Concepcion, Jana Ebler, Arkarachai Fungtammasan, Alexey Kolesnikov, Nathan D Olson, et al. Accurate circular consensus long-read sequencing improves variant detection and assembly of a human genome. Nature biotechnology, pages 1–8, 2019.*

**Second round of review**

**Reviewer 1**

Although I still believe that the authors should present a more comprehensive work (considering the perviously published work by the same group on GB), I agree with the authors that the current form of the mansucript is suitable for a "Short Report", and they have adequately addressed my technical comments.

**Reviewer 2**

The authors have addressed all my previous comments.
